# Identifying Mobile Sensing Indicators of Stress-Resilience

**DOI:** 10.1145/3463528

**Published:** 2021-06-24

**Authors:** DANIEL A. ADLER, VINCENT W.-S. TSENG, GENGMO QI, JOSEPH SCARPA, SRIJAN SEN, TANZEEM CHOUDHURY

**Affiliations:** Information Science, Cornell Tech; Information Science, Cornell Tech; Computer Science, Cornell University; Anesthesiology, Weill Cornell Medicine; Psychiatry, University of Michigan; Information Science, Cornell Tech.

**Keywords:** Human-centered computing → Empirical studies in ubiquitous and mobile computing, Applied computing → *Life and medical sciences*, Computing methodologies → *Artificial intelligence*, mobile sensing, mental health, deep generative models, wearable technology

## Abstract

Resident physicians (residents) experiencing prolonged workplace stress are at risk of developing mental health symptoms. Creating novel, unobtrusive measures of resilience would provide an accessible approach to evaluate symptom susceptibility without the perceived stigma of formal mental health assessments. In this work, we created a system to find indicators of resilience using passive wearable sensors and smartphone-delivered ecological momentary assessment (EMA). This system identified indicators of resilience during a medical internship, the high stress first-year of a residency program. We then created density estimation approaches to predict these indicators before mental health changes occurred, and validated whether the predicted indicators were also associated with resilience. Our system identified resilience indicators associated with physical activity (step count), sleeping behavior, reduced heart rate, increased mood, and reduced mood variability. Density estimation models were able to replicate a subset of the associations between sleeping behavior, heart rate, and resilience. To the best of our knowledge, this work provides the first methodology to identify and predict indicators of resilience using passive sensing and EMA. Researchers studying resident mental health can apply this approach to design resilience-building interventions and prevent mental health symptom development.

## 1. INTRODUCTION

Individuals encounter a variety of stressors within the workplace, and navigating these stressors requires resilience. In 2015, the American Psychological Association found that 65% of Americans believed work to be one of the top two stressors within their lives [4]. In addition, those who work in psychologically demanding environments are more likely to develop depression, anxiety, and substance abuse disorders [23, 73]. Under prolonged stress, individuals may experience a decline in mental health [25, 31] which could be prevented with early-intervention.

Resident physicians, both employees and trainees, work within a psychologically demanding environment, which requires resilience. Resident physicians often work 80 hours per week [6], and experience a variety of emotional stressors while treating patients [62]. This demand may contribute to higher rates of depression (25–33%) [56] among resident physicians compared to graduate students overall, and other young adults within the general population (8–15%) [32]. Ideally, residents could frequently assess their own mental health and seek early-intervention, but residents are unlikely to engage with mental health services due to systemic factors including the perceived stigma around mental health [9, 58], and a lack of time to seek professional mental health support [32]. Thus, stress-mediated interventions often focus on coping mechanisms (eg, exercise) [84], considering these systemic factors.

Finding unobtrusive indicators of resilience may provide a more accessible approach to identify mental health risk without the associated stigma. Researchers have leveraged unobtrusive measurement, using smartphone and wearable devices, to create personalized digital phenotypes of mental health and well-being [15, 38, 90]. Previous work has analyzed the potential for creating digital phenotypes of students [88, 97, 106], schizophrenia patients [99, 100], and more recently employees [57] by combining features derived from passive sensors and short, self-reported survey measures, called ecological momentary assessments (EMA). There is now potential to develop equivalent unobtrusive measures of resilience [59, 67, 69].

The goal of this study was to identify unobtrusive indicators of resilience using data collected from mobile devices. We specifically focused on finding indicators that could be identified or predicted early-on in the internship, and thus be used to design preventive resilience-building interventions. Resilience was measured within a specific population: medical interns (first-year resident physicians) experiencing prolonged workplace stress. Thus, we specifically studied stress-resilience. We would like to state upfront that identifying indicators of resilience for medical interns more broadly has both positive and negative implications. Residents may feel uncomfortable with using passive data-collection technologies to understand their mental health and resilience.

We will review these implications in our discussion.

The contributions for this work are:

1. We developed a system using passive sensing features collected from wearable devices and ecological momentary assessment (EMA) to find indicators of stress-resilience.
2. We used quarterly measures of depression symptoms, collected from medical interns, to identify stress-resilient and stress-sensitive individuals. The stress-resilient individuals experienced minimal depression symptom changes during the internship. Depression symptoms were measured via the nine-item patient health questionnaire (PHQ-9).
3. We then identified specific passive sensing and EMA features that were indicators of stress-resilience, because they were significantly (*𝛼* = 0.5) associated with distinguishing stress-resilient from stress-sensitive individuals. We found significant links between resilience and physical activity (step count), sleeping behavior (seconds of sleep and in bed), heart rate, and daily mood.
4. We developed and validated novel density estimation approaches to predict during-internship stress-resilience indicators from pre-internship data. We then validated if the predicted indicators followed the same associations with stress-resilience as the actual indicators. We found that our generated data accurately replicated a subset of the associations between sleeping behaviors, heart rate, and stress-resilience.
5. We discussed the implications of this work for communities studying resilience, resident well-being, and generative models, as well as the ethical implications of predicting employee mental health more broadly.

## 2. BACKGROUND AND RELATED WORK

### 2.1 Resilience and Mental Health

*Resilience* can be described as a process in which individuals positively respond or adapt to changing circumstances within their lives. Traditionally, when defining resilience, *circumstances* imply an adverse event, or negative life circumstance, that requires some amount of adjustment within an individual [54]. That being said, resilience can be applied to circumstances that individuals face day-to-day [14], rather than a specific adverse event, and also many events that are viewed positively (eg, marriage, a job promotion, beginning school) might require some amount of resilience [22]. Resilience also implies that individuals adapt *positively* to the circumstances they face, which requires context-dependent indicators to describe whether individuals are resilient within a specific situation [53].

There are multiple methods to measure resilience. *Trait resilience* measures describe resilience as a set of personality traits that help individuals adapt to adverse circumstances. Trait resilience is measured using a variety of rating scales, and the outcomes of these scales correlate with mental health symptoms [35]. However, reliability between major trait resilience scales is low [104]. A potential better method to describe and measure resilience is as an outcomes-based process (*process resilience*) [21, 40, 54] that occurs when individuals adapt to minimize the impact of stress. Process resilience can be measured by tracking trajectories of mental health while individuals are under stress [34]. Individuals who do not experience mental health changes over a prolonged period are identified as *stress-resilient*, and this type of resilience tracking has become common in research studying the effects of workplace stress on mental health [26, 31]. For convenience, we will use *resilience*, *stress-resilience*, and *process resilience* interchangeably throughout the rest of this work.

### 2.2 Resident Physicians and Mental Health

*Resident physicians*, part employee and part trainee, are a specific population that experience a variety of situational, personal, and professional stressors throughout the duration of their programs [50]. Residents can experience prolonged sleep deprivation [41, 42, 50], often caused by extremely long shift hours [6, 27], and endure emotional trauma through consistent encounters with fatalities, childhood illness, and chronic disability [16, 62]. After prolonged occupational stress, residents can develop *burnout*, which is described by emotional exhaustion, cynicism, and a sense of self inefficacy [55, 89]. Burnout is dangerous for resident physicians’ mental health, and is associated with increased depression and anxiety [44].

Resident physicians are particularly at risk for changes in mental health during the first year of their program, called a *medical internship*. Research has shown that interns have high levels of depression, anxiety, fatigue, and distress that can persist throughout the duration of their residency programs [7, 94]. There are a number of pre-internship factors that are associated with changes in mental health and well-being during an internship [31, 83], and behavioral changes that occur during the internship may be indicative of future mental health changes [41, 42]. Understanding whether mental health will change early-on could offset the potential effects of prolonged stress if residents are able to build resilient behaviors.

### 2.3 Identifying Methods to Improve Resident Physician Resilience

Introducing resilience-building programs early-on within a medical internship can reduce the impact of stress on mental health. Researchers have proposed resiliency training programs and identified resilience-building behaviors that reduced burnout and improved mental health [62, 109]. That being said, residents often choose not to engage in interventions to improve resilience and mental health [58, 79, 93], citing that they do not have time or access to treatment, they would prefer to self-manage their mental health, and they are concerned about the confidentiality and potential social consequences of seeking external treatment (perceived stigma) [32]. Research suggests that improving sleep and physical activity habits may improve resident mental health and well-being [47, 68], but it may be difficult for residents to modify these behaviors during the internship. Creating unobtrusive measurement systems that can detect resilient behaviors early-on could help residents identify mental health risk factors and take action before symptoms develop. This data could also be anonymized and aggregated to guide program directors towards structural interventions (eg, increased schedule flexibility) that improve mental health.

### 2.4 Unobtrusive Mental Health Monitoring Using Passive Sensors and EMA

*Passive sensing* along with *ecological momentary assessments* (EMA) delivered through a smartphone application (i.e. *mobile sensing*) can be used to predict trajectories of mental health and well-being. A passive sensor is any sensor that can collect data with little-to-no human interaction. EMAs are *in-the-moment* assessments, often delivered digitally, used to collect more frequent measurements of mental health outside of a clinic [30, 74, 86]. Thus, both passive sensing and EMA are unobtrusive on-device measurements. Previous studies leveraged smartphone sensors, wearables, and EMAs to find significant correlations between the collected mobile sensing data and mental health [15, 42, 61, 82, 88, 97, 98]. These technologies can also be used to predict trajectories of serious chronic mental illness, including bipolar disorder [24], schizophrenia [2, 8, 11, 12, 99, 100, 102], and depression [80, 81, 101].

In this work, we found unobtrusive indicators of medical intern stress-resilience using passive sensing and EMA. We specifically focused on identifying indicators using data collected prior to the internship, or predicted from data collected prior to the internship, because this information could guide interns and program directors towards effective and targeted mental health symptom prevention strategies.

## 3. THE INTERN HEALTH STUDY AND DATASET

The Intern Health Study is an ongoing multi-site prospective cohort study to understand the links between behaviors, mental health, and well-being as resident physicians adapted to the stress of their programs. The first year of residency, also called a medical internship, is known to impact resident mental health and well-being [7, 94]. Participating sites were located across the United States, and a full list of participating sites can be found on the study websites [48, 87].

Interns starting their residency at a participating site were eligible to enroll online. After consenting to the study, participants were mailed a Fitbit Charge 2 [18] for passive behavioral and physiological tracking, and completed a baseline assessment via a smartphone study application 1–2 months prior to the commencement of the internship. In addition, the study application sent notifications to complete daily mood ecological momentary assessments (EMAs), and facilitated data transfer from the Fitbit to a secure storage platform. Participants were asked to participate in Fitbit tracking and complete daily EMAs beginning 1–2 months prior to their internship through the end of the internship (~14 months total). Lastly, participants completed quarterly mental health assessments for depression symptoms at internship months 3, 6, 9, and 12, to further gauge how they adapted to their new work. Table 1 summarizes the passive sensing and EMA data.

This study was approved by the University of Michigan Institutional Review Board (IRB) and all subjects provided informed consent after receiving a complete description of the study. The collected data was used for research purposes only. Participants were incentivized to participate by receiving the Fitbit device and up to US $125, distributed five times throughout the year (US $25 each time) with continued participation.

### 3.1 Passive Wearable Sensing

Participants were mailed a Fitbit Charge 2 [18]. The Fitbit device continuously tracked minute-by-minute step count, heart rate, whether a participant was sleeping, and the type of sleep. Prior research has examined and determined that Fitbits are an accurate consumer product for tracking sleep, activity, and heart rate for research purposes [17, 60, 63]. Information about how Fitbit devices track heart rate, steps, and infer sleep states is available on the Fitbit website, but is limited due to the proprietary nature of Fitbit’s algorithms [19, 20]. We will briefly describe what is known below.

Fitbit uses a three-axis accelerometer to infer step count information. To detect heart rate, LED lights installed on the bottom of the Fitbit flash many times per second, and light-sensitive photodiodes then detect volume changes within wrist capillaries to infer heart rate beats per minute (BPM). Lastly, Fitbit combines the accelerometer and heart rate information to infer when an individual is sleeping, by measuring when an individual has stopped moving for one hour, and then measuring changes in heart rate to infer the sleep stage. The Fitbit application programming interface (API) outputs two different sleep categorizations, and a query to the API may respond in a mix of the two categorizations. The *classic* categorization uses the accelerometer to infer general sleep categories (asleep, restless), and the newer *stages* categorization uses the accelerometer and heart rate monitor to infer sleep stages (deep, light, rapid eye movement). The Fitbit also collects data on short wake cycles (<3 minutes) that occur between sleep.

### 3.2 Mood Ecological Momentary Assessment (EMA)

Ecological Momentary Assessments (EMAs) are a standard method for assessing in-situ mental health and well-being [30, 97, 99]. EMAs were completed once per day by participants through a smartphone application created for this study at a participant-designated time between 5PM and 10PM. Please refer to the Intern Health Study website for more information on the study application [87]. The EMA contained one question that asked participants to rate their daily average mood from 1 (low) to 10 (high).

### 3.3 Baseline and Quarterly Assessments

Participants completed baseline (BL) and quarterly (Q1–4) assessments upon beginning their internship that contained questions regarding demographics, including age, sex, ethnicity, and also information on their medical specialty. In addition, at baseline and at the end of each quarter, interns completed the nine-question patient health questionnaire (PHQ-9), a self-reported measure for depression symptoms [46]. A higher PHQ-9 score indicates a higher severity of depression symptoms. A variety of other survey measures were taken baseline and quarterly, but were not used in this work. Please refer to the Intern Health Study website [87] for more details.

We chose to not use the demographic variables or specialty information as indicators of resilience, but these variables were used as controlled covariates in some analyses throughout this work. We decided that we did not have enough contextual information to understand individuals’ circumstances that may explain why demographic variables were related to resilience. We discuss potential methods to account for demographics within mental health models in section 7.6.

### 3.4 Feature Creation

Features were created from the Fitbit passive sensing and EMA data collected. We now briefly describe each feature in more detail. A summary of features can be found in Table 2.

#### 3.4.1 Heart Rate.

The Fitbit tracks minute-by-minute heart rate. We computed the mean hourly heart rate for each hour and participant. We chose to use the mean instead of the median as a summary feature, as we believed extreme heart rate values recorded within an hour would be captured by using the mean as our summary statistic. Heart rate variability, which can be used as an indicator for stress [43], was not available for all participants within our dataset, and was not used within this study. We hope to analyze heart rate variability and its relationship to intern stress within future research.

#### 3.4.2. Daily Mood Ecological Momentary Assessment (EMA).

Participants were notified to complete a daily mood EMA through the study smartphone application. Modeling sensor data sampled at different frequencies is an active area of research. For simplicity, we used local interpolation to approximate an hourly mood EMA from the daily mood EMA. Prior work found that local interpolation of irregularly sampled features in time series still preserves key characteristics of the original time series [45]. The interpolating procedure follows.

If a mood EMA was completed on a given day, we filled the hours of that day with the EMA value, from the time the participant woke up from a previous sleep cycle, up to the time when the participant woke up following the next sleep cycle that was greater than two hours. Using sleep cycles as start and end points for the interpolation procedure allowed us to capture the spirit of the mood EMA question prompt, which asked participants for their average mood over the entire day (see Table 1). We expected most participants would answer this question thinking back to when they woke up that day. If multiple mood EMAs were recorded on a day (implying the participant completed the survey more than once), the average of the mood EMAs was taken, and this average value was used for interpolation. Mood EMAs were filled up to 24 hours after the EMA was completed.

Similar to previous work, we added random noise *ϵ ~ Uniform* (0, 0.2) to each mood EMA so that we could model mood as a continuous variable [105]. 0.2 was chosen, because after averaging daily mood values, mood EMA values existed on a scale from 1–10 with 0.5 increments. Adding noise ≤0.2 allowed us to create continuous values, while preserving a gap between neighboring discrete mood values so they could be recovered.

#### 3.4.3 Sleep.

The Fitbit algorithm categorizes the type of sleep (see section 3.1), and records short wake cycles that occur in-between sleep. For simplicity, we aggregated the recorded time sleeping within an hour (within any type of sleep), and the total number of seconds in bed (which includes both sleep and short wake cycles). Note that if a sleep cycle extends beyond an hour, the Fitbit may record one long multi-hour sleep cycle.

#### 3.4.4 Steps.

The Fitbit tracks minute-by-minute step counts. We summed through all steps taken within an hour to create an hourly step count feature.

### 3.5 Data Cleaning

#### 3.5.1 Missing Data.

After creating the initial hourly features from raw data, we analyzed the data for missing values and outliers. The following types of missing data were identified, with mitigation procedures:

- For step and sleep features, we identified hours that contained classified sleep, but no recorded steps, and vice-versa. We deemed that these hours could be considered “non-random missing data”, which assumed, for example, that the reasoning behind the missing sleep values was due to an individual being awake. Given this assumption, missing data for step and sleep features were filled with 0s during hours where either of these cases occurred.
- After creating the interpolated mood EMA, we dropped all remaining data points without a mood score. This occurred when a mood score had not been recorded within the past 24 hours.
- Heart rate data should be continuously recorded by the Fitbit. We dropped hours that did not contain any heart rate data for a participant.

#### 3.5.2 Outlier Filtering.

After dropping missing values, we identified multivariate outliers within the hourly features listed in Table 2. Prior work using mobile sensing data to model mental health included methodological choices that reduced model sensitivity to outliers [99, 100]. There were two potential types of outliers within this work: (1) extremely unrealistic sensor values (eg, walking 20,000 steps in a single hour) within the study population, or (2) outliers that have an implication for mental health (eg, long sleep duration). We aimed to filter outlier type (1), but not outlier type (2).

Outliers were filtered using an Isolation Forest [52] algorithm. Isolation forests recursively partition data through randomly selected features. A set of partitions can be described as a path to a set of samples, and samples that are partitioned by shorter paths are classified as outliers. We created an Isolation Forest using the scikit-learn library [70], with 250 trees, and randomly partitioned samples into each tree. The maximum number of features per tree was set to the length of the feature space.

The results of outlier filtering are summarized in Table 3. Note that some features, such as the seconds of sleep per hour, are likely to have majority “0” values because individuals spend most of their hours awake. 73,722 samples (2.9% of the total samples) were classified as outliers, and removed. We would like to note that it is possible some type 2 outliers were likely filtered during this process, which may affect our ability to find indicators of resilience in this work. We provide further analysis to investigate the impact of outliers on distinguishing stress-resilient from stress-sensitive individuals in section 4.2.

#### 3.5.3 Filtering Out Participants with Extremely Low Data Quality.

Similar to previous work in mobile sensing for mental health prediction [2, 92], we required participants to have a minimum number of hours of data collected for training prediction models. We filtered out study participants that did not have at least 100 total hours of data prior to the internship starting, and during the internship year. Though it is possible that low data availability for a participant could have an implication for mental health, we still believed that having a minimum threshold was important for modeling. To account for the effect of low data on mental health, we added an additional feature for analysis to account for low data quality, described in the following sections. We lastly filtered out individuals who had an hourly feature variance of zero, for any hourly feature. A summary of the data filtering procedure can be found in Table 4.

The objective of this work was to find indicators of resilience when individuals experienced internship stress. Ideally, we would have predicted changes that occurred at each quarter of the internship and developed a more fine-grained notion of when individuals were resilient. As the internship progressed, the availability and quality of participant data, specifically after the second quarter of the internship (see Table 4), decreased. Thus, we focused on a simpler task, and grouped the hourly features together before the internship into a single multivariate baseline distribution per participant, and the hourly features during the internship (Q1 to Q4) as another multivariate internship distribution per participant. We then created indicators from these two distributions, described below.

### 3.6 Indicator Creation

**Fig. 1.**
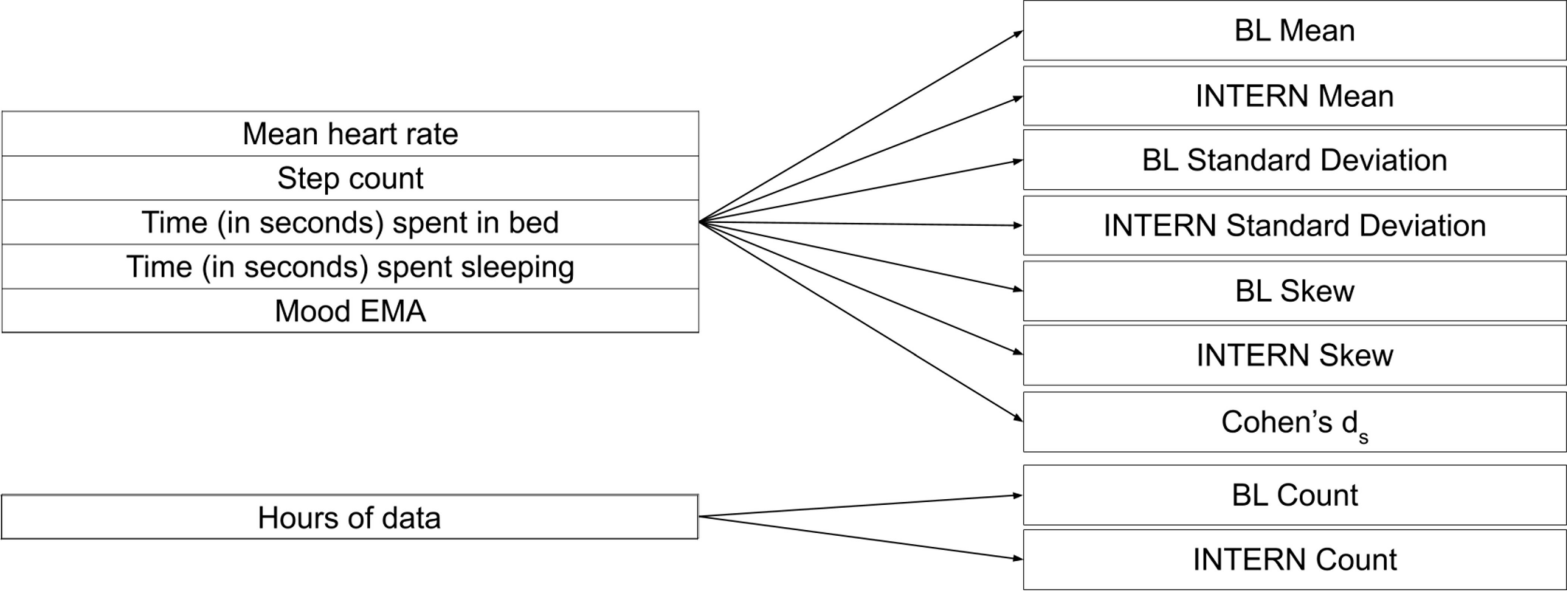
Summary of the 37 different indicators used in this work. Values are either specific to the period before (BL), during (INTERN) the internship, or captured a difference in a specific metric between the INTERN and BL periods (Cohen’s *d*_*s*_). The indicators on the right are calculated for each metric listed in the same section on the left. For example, we calculated the Mean, Standard Deviation, and Skew for both the BL and INTERN periods, as well as the Cohen’s *d*_*s*_ for the mean heart rate. This results in 7 total indicators for the mean heart rate, and this process can be repeated for each of the 5 hourly features (35 indicators). 2 additional indicators were created to capture information about missing data, specifically the count of data per participant in the BL and INTERN periods, resulting in 37 total indicators.

We developed 37 different passive sensing and EMA indicators from the hourly features based upon multiple characteristics from the multivariate baseline and internship distributions. A summary of the indicators can be found in Figure 1. Similar indicators were used in prior work measuring the effect of mobile data on mood [42]. Let us define the multivariate baseline distribution of hourly features for an individual as *A*, and the multivariate internship distribution of hourly features for an individual as *B*. Suppose we have *m* features, and defining *j* ∈ {1, ...*m*}, *A*_*j*_ and *B*_*j*_ are the distributions for each hourly feature per participant. We computed the mean and standard deviation of the hourly features in both the baseline $\left({\bar{X}}_{A_{j}},SD_{A_{j}}\right)$ and internship $\left({\bar{X}}_{B_{j}},SD_{B_{j}}\right)$ distributions.In addition, to create a measure of missing data, we computed the number of hours of total data collected for both the baseline (*n*_*A*_) and internship (*n*_*B*_) periods.

We computed the empirical skew for each feature in both the baseline internship distributions, which is a measure of how “balanced” a distribution is. We expected many of the features in our dataset, such as the mood EMA, to be non-gaussian. We initially created nonparametric indicators (eg, median and interquartile range), but found these statistics were highly correlated with their parametric counterparts. Thus, the skew indicator per feature was used to capture how the non-gaussian nature of each feature distribution was associated with stress-resilience. We used the Pearson’s skew coefficient [95], which measures the difference between the empirical mean $\left(\bar{X}\right)$ and the median (*v*) divided by the standard deviation (*SD*). For example, for the baseline distribution, a multivariate data point with *m* features, and a single hourly feature (*A*_*j*_) for an individual:

(1) $$Skew_{A_{j}}=\frac{3\left({\bar{X}}_{A_{j}}-v_{A_{j}}\right)}{SD_{A_{j}}}\;\;\;j\in \{1,\ldots ,m\}$$

Finally, we computed the standardized difference in means, or Cohen’s *d*_*s*_, between the baseline and internship period, for each hourly feature. The formula for the Cohen’s *d*_*s*_ is found in equation 2. We will refer to the vector of Cohen’s *d*_*s*_ for each feature as *d*_*s*_, and the Cohen’s *d*_*s*_ for each feature as *d*_*sj*_ . For each feature, this is computed as follows:

(2) $$d_{s_{j}}=\frac{{\bar{X}}_{B_{j}}-{\bar{X}}_{A_{j}}}{\sqrt{\frac{\left(n_{A}-1\right)SD_{A_{j}}^{2}+\left(n_{B}-1\right)SD_{B_{j}}^{2}}{\left(n_{A}+n_{B}-2\right)}}}\;\;j\in \{1,\ldots ,m\}$$

## 4 EVALUATION: IDENTIFYING INDICATORS OF RESILIENCE

Table 5 shows a summary of the data used for analysis after cleaning and outlier filtering. Table 6 displays demographic information from the analysis cohort compared to sex and race/ethnicity 2018–19 statistics of graduating medical students from the Association of American Medical Colleges (AAMC) [65, 66]. Significant differences were found between the analyzed data and AAMC demographic information for both sex (*χ*^2^ = 12.46, *P* < .001) and race/ethnicity (*χ*^2^ = 35.90, *P* < .001). Table 7 describes the medical specialties of interns within the dataset. Specialties were not compared to AAMC specialty statistics, as we only captured a subset of specialties and thus categorical comparison was difficult.

### 4.1 Identifying Stress-Resilient and Sensitive Participants

Resilience is defined as adaptation to circumstance. We looked to identify a set of individuals within the population whose depression symptoms changed minimally throughout the internship. Previous studies [25, 31] labeled population subsets that experienced minimal mental health changes as the “stress-resilient” population. By identifying this population, we could then find passive sensing and EMA indicators that distinguished stress-resilient and stress-sensitive individuals. We used quadratic growth mixture models (GMMs) [75] to identify distinct trajectories of depression symptom changes across the population, measured using recorded PHQ-9 changes during baseline and the internship. Previous work used GMMs to identify mental health trajectories distinguishing stress-resilient from stress-sensitive individuals [25, 31]. GMMs are similar to linear mixed-effects models, but the key difference is that GMMs identify distinct latent classes within a dataset, and fit a curve to each of these distinct classes. Expectation-maximization is used to optimize both the model parameters and fit classes across individuals as a latent variable [76]. Quadratic models were chosen over linear models, as previous studies modeling resilience within medical interns [31] found that depression symptoms increased when individuals experienced stress, and decreased after a period of time.

**Fig. 2.**
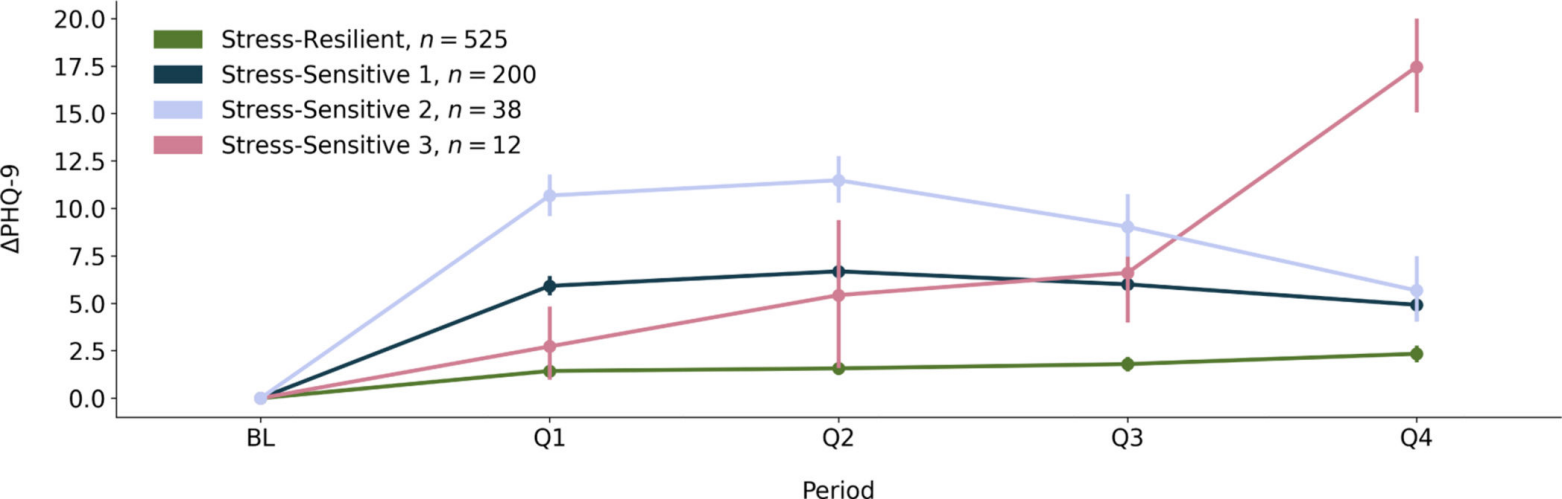
The resulting trajectories from the 4-class quadratic growth mixture model. Each curve represents the change in depression symptom trajectory for the subset of the population within that class. Points represent the mean Δ*PHQ* − 9 (change in depression symptoms) for that period and population subset represented by that trajectory, and error bars are a 95% confidence interval around the mean. The y-axis, Δ*PHQ* − 9, are the changes in depression symptoms compared to baseline (BL). The x-axis describes the period in which depression symptoms were measured, including the baseline period before the internship (BL), and each quarter, or 3-month period (Q1–4), of the year-long internship. One class was labeled the “Stress-Resilient” class, because it contained a subset of the population who experienced minimal changes in depression symptoms throughout the internship. The legend shows the labels for each class, as well as the size of the population subset (n) the trajectories represent.

We experimented with identifying 2–5 distinct classes within our dataset. We then chose the number of classes that minimized both the Akaike Information Criterion (AIC) and the Bayesian Information Criterion (BIC). The resulting AIC and BIC for each pre-defined number of classes can be found in Table 8. We found the 4-class model minimized the AIC (17,127) and BIC (17,215). The depression symptom change (Δ*PHQ* − 9) trajectories from the 4 class model can be found in Figure 2. The majority class (*n* = 525, 68% of participants) who experienced minimal PHQ-9 (depression symptom) changes was qualitatively determined to be the “stress-resilient” population, and the combined other classes (*n* = 250, 32% of participants) were determined to be the “stress-sensitive” population. We acknowledge that the stress-sensitive population combined the trajectories from 3 distinct classes, and we plan to analyze distinct stress-sensitive classes in future work.

### 4.2 The Impact of Outliers on Identifying Stress-Resilient Participants

We analyzed if our outlier filtering procedure affected our ability to distinguish stress-resilient versus stress-sensitive individuals. If outlier values were characteristic of stress-sensitivity, we would expect that a higher number of outliers would be filtered out for stress-sensitive compared to stress-resilient participants. A Shapiro-Wilk test showed that the outlier count distribution across participants was non-normally (*P* < .05) distributed. A Mann-Whitney *U* test was performed to examine if the number of outliers identified across stress-sensitive participants was significantly greater than the number of outliers identified across stress-resilient participants. The test was non-significant (*U* = 69, 510.5, *P* > .05). We also confirmed that participants were not entirely filtered out of our dataset during the outlier removal procedure. Thus, we believe these outliers did not contain information that distinguished stress-resilient from stress-sensitive individuals.

### 4.3 Identifying Passive Sensing and EMA Indicators of Resilience

#### 4.3.1 Generalized Estimating Equations (GEE).

We used generalized estimating equations (GEE) [33, 100] to find which indicators, defined in Figure 1, significantly differentiated stress-resilient and stress-sensitive individuals. We initially used logistic regression, a simpler generalized linear model, but the regression failed to identify indicators that distinguished stress-resilient from stress-sensitive individuals. GEE is a type of linear model that can be applied to measure population effects on clustered or grouped data, and GEE can be more robust compared to other grouped linear models such as linear mixed-effects models because GEE requires less assumptions on the underlying data distributions [37]. Sex and age were controlled for within each model. Sex and age were chosen as controls because we believed these are two characteristics that an individual might be more comfortable to share with an implemented resilience-measurement system, compared to a characteristic like ethnicity, or baseline depression status. We used the internship specialty as the grouping variable, because the intensity of work can vary by specialty [6]. Continuous indicators were standardized by subtracting the mean and dividing by the standard deviation prior to conducting the regression. In addition, a constant term of “1” was added to the regression model as a y-intercept.

A summary of the indicators used within the GEE can be found in Figure 1. We first created a set of “univariate GEE” models with each potential indicator isolated and controls, to first find which features significantly differentiated stress-resilient and sensitive individuals. We then conducted a “multivariate GEE” where we modeled the significant indicators and controls together, after removing indicators that were highly correlated. More information on identifying and removing correlated indicators can be found in Appendix I, within the supplementary material.

#### 4.3.2 GEE Results.

The univariate and multivariate GEE results are listed in Table 9. A positive GEE *β* coefficient shows a positive association between an indicator and the likelihood an individual is stress-resilient with all other independent variables held constant. The magnitude of the *β* coefficient can be interpreted as the strength of the association.

Out of the 37 potential indicators, 17 were significantly associated with stress-resilience within the univariate GEEs. For space, we only describe the most significant (*P* < .001) indicators within the text. Having a higher average number of seconds in bed and sleep during the internship increased the likelihood of resilience. Hourly sleep distributions are skewed (most individuals are not sleeping during the day). Increasing the skew translates to the tail of the distribution (more hours with higher seconds of sleep) becoming larger, and this increase in skew during the internship increased the likelihood of stress-resilience. The mood EMA feature showed a number of significant associations with stress-resilience, which was expected given low mood is a direct symptom of depression on the PHQ-9 [46]. A higher mood score during the baseline and internship, as well as lower fluctuations in mood (decreased standard deviation) increased the likelihood of stress-resilience. An increased mood score (positive Cohen’s *d*_*s*_) increased the likelihood of stress-resilience.

After removing correlated features, 3 indicators were included in the multivariate GEE model. The 3 indicators were the INTERN step count skew, seconds in bed Cohen’s *d*_*s*_, and mood EMA Cohen’s *d*_*s*_. The high number of filtered indicators showed that the potential indicators were highly correlated. All 3 indicators were significantly associated with resilience, and we describe them further. The step count distributions are skewed because there are many hours during the day when an individual is not moving (hourly step count = 0). Thus, decreasing this skew shifts the mode of the distribution away from 0, i.e. there are more hours spent with nonzero step counts. This decrease during the internship period increased the likelihood of stress-resilience $\left(\beta_{M}=-0.16,P_{M}<.01\right)$. Increasing the amount of time spent in bed increased the likelihood of stress-resilience $\left(\beta_{M}=0.11,P_{M}<0.05\right)$, as well as increasing one’s mood $\left(\beta_{M}=0.26,P_{M}<0.05\right)$.

## 5. PREDICTIVE MODELING APPROACH

### 5.1 Motivation

The results in Table 9 showed that there were a variety of indicators that summarized both the baseline and internship hourly feature distributions and were significantly associated with distinguishing stress-resilient versus stress-sensitive individuals. An application of this analysis would be to use the found indicators to guide interns towards wellness interventions [47, 68], or help residency program directors create interventions that improve resilience. Interns may be more willing to engage in these interventions before they are time-constrained by their residency program, and are impacted by internship stress. For example, if the system indicates an individual is less likely to engage in physical activity during the internship, which is linked to higher stress-sensitivity, an intern could begin to build exercise goals into their routine before the internship begins [47].

13 out of 17 of the found indicators were associated with mobile data collected during the internship. These indicators are unknown during the baseline period. We aimed to predict these indicators using mobile data collected during the baseline period, which would be needed for early-assessment and intervention. We first experimented with regression models, including random forests, gradient boosting trees, and multilayer perceptrons, to approximate the resilience indicators from baseline data. We found that these models were unable to achieve accurate predictions across all indicators.

**Fig. 3.**
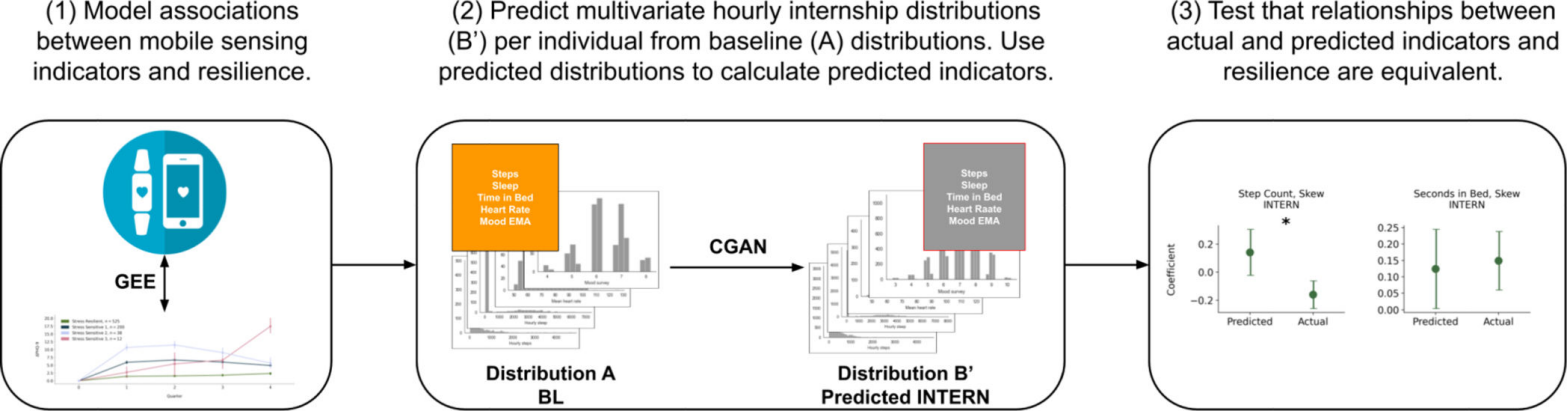
The full analysis pipeline in this work. We let *A* be the multivariate hourly baseline (BL) feature distributions per-individual, and *B*′ be the predicted multivariate hourly internship (INTERN) feature distribution per-individual. (1) We first found relationships between the actual mobile sensing indicators using both the baseline and internship data and resilience (see Table 9). We then built conditional generative adversarial networks (CGANs) to predict the internship data (*B*′) from the baseline data (A) per-individual. We calculated predicted mobile sensing indicators using both *A* and *B*′. Lastly, in (3),we validated whether the associations between the predicted indicators and resilience were equivalent to the relationships between the actual indicators and resilience (see Table 12 and Figure 7).

We thus pivoted our analysis to a more complex approach, specifically using density estimation techniques, to predict a multivariate distribution of the hourly features per-individual. Per these multivariate distributions, we would be able to calculate a set of predicted resilience indicators, and verify whether the relationships between the predicted indicators and resilience aligned with the actual indicators and resilience. Figure 3 summarizes our analysis.

### 5.2 Overview of Density Estimation Models Used in this Work

We now give an overview of the density estimation techniques used to generate the during-internship multivariate hourly feature distributions from the multivariate hourly baseline distributions per participant. More specific details, including the equations, architecture, and hyperparameters for each model can be found in Appendix II within the supplementary material.

#### 5.2.1 Conditional Generative Adversarial Networks (CGAN).

We specifically chose to use generative adversarial networks (GANs) for predicting the multivariate internship distributions *B* from the multivariate baseline distributions *A*. We decided to use this approach because GANs can generate high quality samples of complex data distributions [29] compared to simpler density estimation approaches (eg, kernel density estimation), and there is a large amount of previous work using GANs in a supervised format to predict a specific distribution from an input distribution [3, 39, 108]. GANs are also easier to optimize compared to other generative models (eg, variational autoencoders), because GANs do not attempt to approximate intractable likelihoods [29]. GANs are a type of deep learning model, and the models we used were based upon different encoder-decoder neural networks, similar to [39].

In this work, we wanted to generate a specific multivariate hourly feature internship distribution *B*, from a multivariate hourly feature baseline distribution *A*, per participant. There is a family of GANs, called conditional GANs (CGAN), that are used to teach a GAN to generate distributions in a supervised manner. The input to the CGAN was a multivariate hourly baseline data point, for a participant $a\in A,a\in R^{m}$ (assuming we have *m* features), and the output was a generated multivariate hourly internship data point $b^{\prime }\in B^{\prime },b^{\prime }\in R^{m}$. After inputting multiple different baseline hourly data points, *A* for a participant, the CGAN can generate a multivariate hourly internship distribution *B*′ for the same participant. Distribution characteristics from the actual *B* and generated *B*′ multivariate hourly distributions can then be compared to assess density estimation performance.

#### 5.2.2 Multitask CGANs.

Multitask learning (MTL) is a machine learning technique used to train separate, but related prediction tasks together [13]. Previous work [88, 92] using mobile sensing data to predict mental health

leveraged MTL to improve model performance. We experimented with MTL approaches based upon the following two assumptions:

1. Data generation for each hourly feature is a separate, but related prediction task. Adding separate neural network output layers for each feature could improve the data generation performance for each feature.
2. Participants experienced a variety of feature changes when beginning the internship, but training a model for each participant would result in overfitting, and not generalize to unseen individuals. MTL can prevent this overfitting by training individual-level models together.

We created three new multitask learning models based upon the CGAN framework. These models were composed of fully connected neural networks, and similar to [88], the models had input “shared layers” where neural network parameters were shared across tasks, and output “single-task” layers, where parameters were specific to each task:

1. **Feature Multitask Learning CGAN (F - CGAN)**: We treated predicting each internship hourly feature distribution (eg, step count, mood EMA) as a separate, but related, prediction task.
2. **Participant Multitask Learning CGAN (P - CGAN)**: We treated clusters of individuals who experienced similar feature changes when they began the internship as a separate task. We first identified clusters of participants who experienced similar feature changes, by clustering participants based upon their Cohen’s *d*_*s*_. We experimented with different clustering approaches and varied the number of clusters. The clustering with the highest silhouette score was chosen (see Appendix II within the supplementary material).
3. **Feature and Participant Multitask Learning CGAN (FP - CGAN)**: We integrated both feature and participant multitasking into one model. In this model, output single-task layers were specific to each feature and cluster.

### 5.3 Training and Testing Procedure

All predictive models were trained using data from 80% (*n* = 611) of the study participants, and we used a form of leave-subject-out validation [99, 100] to report each model results with different hyperparameter choices for a held-out 20% (*n* = 154) of participants. The results from the 20% hold-out set simulate the prediction accuracy of predicting data from new participants who have recently joined the study. For simplicity, we will refer to the 80% training set as the “training” data, and the held-out 20% dataset as the “test” data. An overview of the training and testing data can be found in Table 10. Hyperparameter choices for each model are further explained in Appendix II, within the supplementary material. Clusters for participant multitasking models were created using both baseline and internship training data. For test participants, we predicted data for each cluster, and selected the cluster that minimized the error between the predicted and actual internship first quarter (Q1) data distributions.

In addition to the CGANs, we created two baseline density prediction models, and two baseline multilayer perception regression models. The first model was a density prediction model composed of a fully connected neural network (not a CGAN) trained to input an hourly baseline data point, and output an internship data point (GEN), and the second model was the GEN model with separate participant multitasking output layers (P - GEN). The baseline multilayer perception regression models predicted each feature’s Cohen’s *d*_*s*_ from the baseline mean of the hourly features. We created a model with (P - MLP) and without (MLP) separate participant multitasking output layers. We show the baseline MLP models to predict the Cohen’s *d*_*s*_ as examples, but other baseline models with similar architectures could be trained to predict the variety of resilience indicators described in Table 9.

### 5.4 Evaluation Metric

We calculated the skipped correlation (r∈[−1,1]) between the actual and predicted Cohen’s *d*_*s*_ for each hourly feature to measure model performance. The skipped correlation is less sensitive to bivariate outliers compared to other correlation coefficients (eg, Pearson’s, Spearman’s), which often overestimate model fit [71, 78, 103]. We measured model performance using the Cohen’s *d*_*s*_ because it captures both the central tendency (mean) and variation (standard deviation) within the baseline and generated distributions, and is familiar to both technical and clinical audiences [2, 42, 49]. In addition, the majority of indicators within the multivariate GEE (Table 9) were calculated using the Cohen’s *d*_*s*_. We also evaluated the squared error between the predicted Cohen’s *d*_*s*_ for each model, and the squared error calculated by assigning each test participant the average Cohen’s *d*_*s*_ value within the training data. The squared error calculated using the average Cohen’s *d*_*s*_ was the baseline error in our analysis, and we analyzed if our model error was significantly lower than the error using the average training Cohen’s *d*_*s*_ for each feature.

## 6 EVALUATION: PREDICTING INDICATORS OF RESILIENCE

In this section, we evaluate whether the generative models were able to predict indicators of resilience. We first evaluate the generative models’ performance on the held-out test data, and then we validate whether the predicted indicators calculated using the generated data hold the same associations with resilience as the actual indicators.

**Fig. 4.**
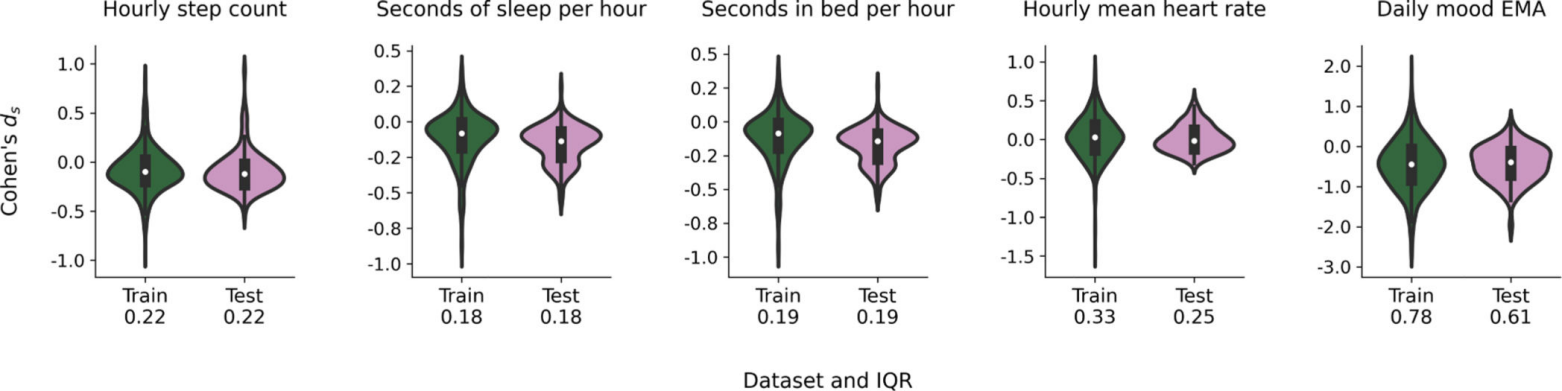
The distribution (histograms) of Cohen’s *d*_*s*_ for training (*n* = 611) and testing (*n* = 154) data. Within each histogram, the boxplots show the median and interquartile range (IQR) of each Cohen’s *d*_*s*_. The numbers below the x-ticks are the IQR of the Cohen’s *d*_*s*_ for the specified dataset.

Figure 4 shows the Cohen’s *d_s_* distributions for each feature split into the train (*n* = 611) and test (*n* = 154) sets. We highlighted the interquartile ranges of each feature’s Cohen’s *d*_*s*_, which were considerably larger in the training data for the hourly mean heart rate (0.33) and daily mood EMA (0.78) compared to the step count (0.22), seconds of sleep (0.18), and seconds in bed (0.19) per hour.

### 6.1 Clustering for Participant Multitask Learning Models

**Fig. 5.**
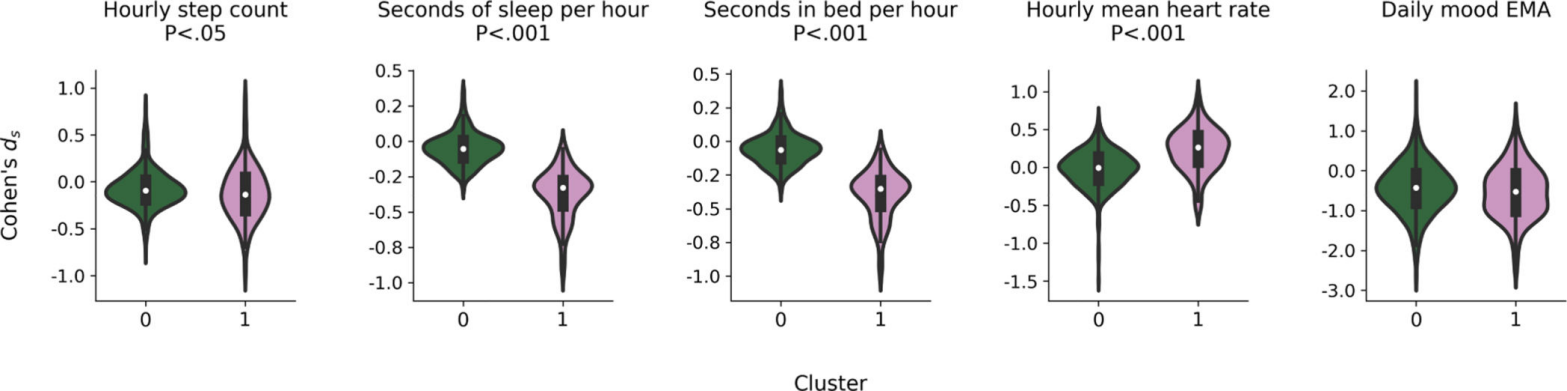
The distribution (histograms) of Cohen’s *d*_*s*_ for the two clusters created for participant multitasking models within the training data. Cluster 0 contained *n* = 510 participants and cluster 1 contained *n* = 111 participants. Within each histogram, the boxplots show the median and interquartile range (IQR) of each Cohen’s *d*_*s*_. The *P* values listed above each boxplot are the result of either a two-sample t-test or Mann-Whitney *U* test with the null-hypothesis that the feature distribution centers of each cluster were equal.

We clustered training participants using their Cohen’s *d*_*s*_ to create each task (each cluster) for the participant multitask learning models. The clustering that achieved the highest silhouette score (0.32) used Agglomerative Clustering with four principle components (using principal components analysis for noise-reduction), and resulted in two clusters with 510 and 111 training participants in each cluster respectively. Figure 5 shows the training data distributions of the passive sensing features and EMA split by cluster. We conducted either an independent two-sample t-test or Mann-Whitney *U* test, with the null hypothesis that the feature means between the two distributions were equal. A Mann-Whitney *U* test was used if a Shapiro-Wilk test revealed that the Cohen’s *d*_*s*_ distribution for a feature was non-normally distributed (*P* < .05). We found significant differences between the feature means for the hourly step count (*U* = 24, 998, *P* < .05), seconds of sleep per hour (*U* = 1, 989, *P* < .001), seconds in bed per hour, (*U* = 1, 991, *P* < .001), and hourly mean heart rate (*U* = 13, 135, *P* < .001). There were no significant differences between clusters for the daily mood EMA.

### 6.2 Model Performance

We calculated the actual and predicted Cohen’s *d*_*s*_, using each predictive model, across test participants and feature. We then calculated the skipped correlation coefficient (r∈[−1,1]) [103] and correlation significance between the actual and predicted Cohen’s *d_s_*. The resulting *r* values are found in Table 11. The FP - CGAN model had both significant (*α* = 0.05) and relatively high correlations between all features, with values of (*r* = 0.31, *P* < .001) for the hourly step count, (*r* = 0.50, *P* < .001) seconds of sleep per hour, (*r* = 0.49, *P* < .001) seconds in bed per hour, (*r* = 0.21, *P* < .05) hourly mean heart rate, and (*r* = 0.37, *P* < .001) for the daily mood EMA.

In addition, we compared the squared error between the predicted and actual Cohen’s *d_s_* for each participant and model to a baseline squared error achieved by assigning each individual the average Cohen’s *d_s_* for each feature from the training data. Shapiro-Wilk tests revealed that each feature error distribution was non-normally distributed (*P* < .05). We conducted a Wilcoxon signed-rank test to assess if the squared errors using the models were significantly less (*P* = 0.05) than the error achieved by assigning the average Cohen’s *d_s_*. Within the highest performing model (FP - CGAN), we found that the errors were significantly less for the seconds of sleep (*W* = 4, 066, *P* < .001) and seconds in bed (*W* = 4, 030, *P* < .001) features.

### 6.3 Comparing CGAN Performance to Other Models

**Fig. 6.**
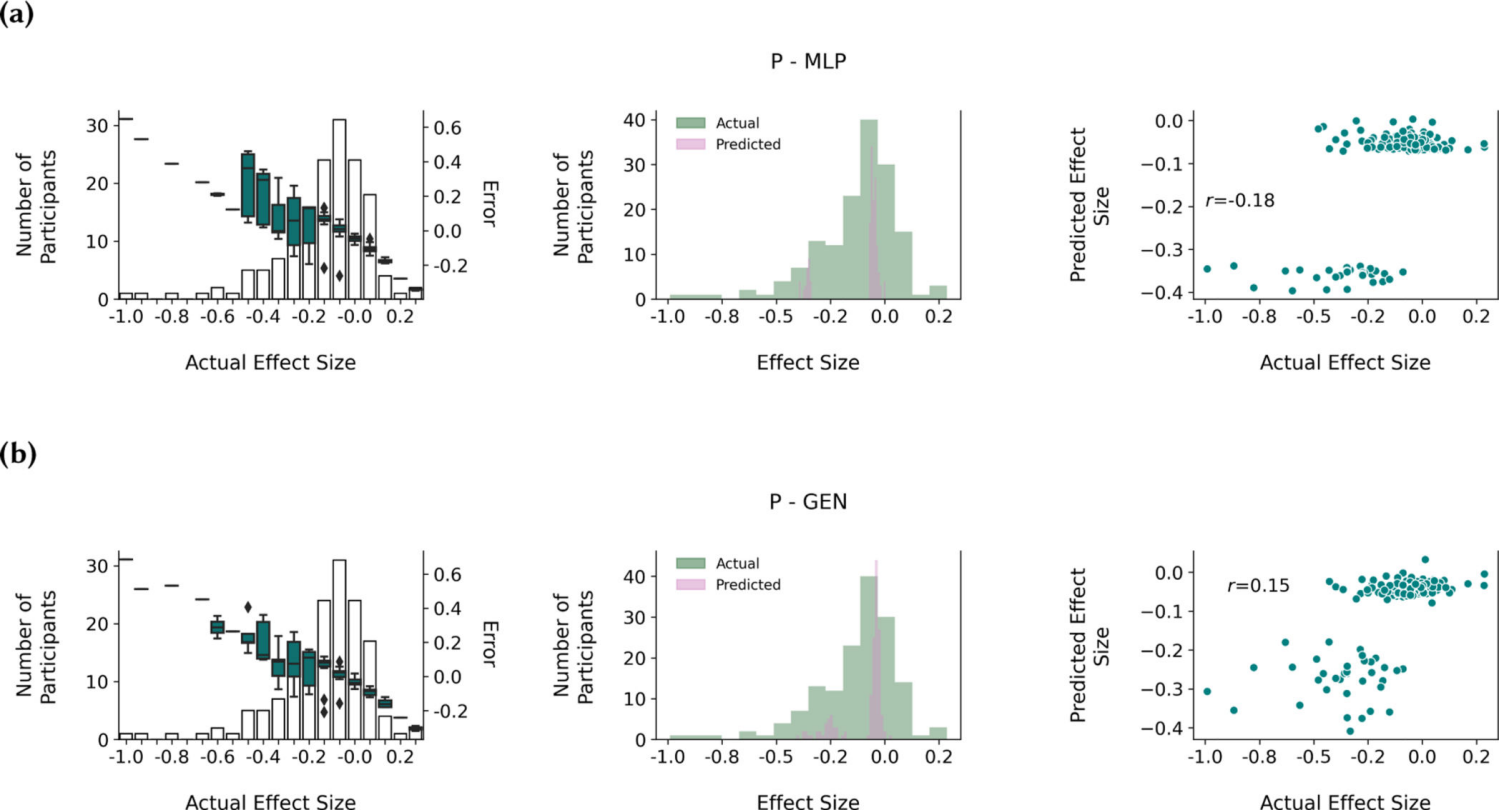

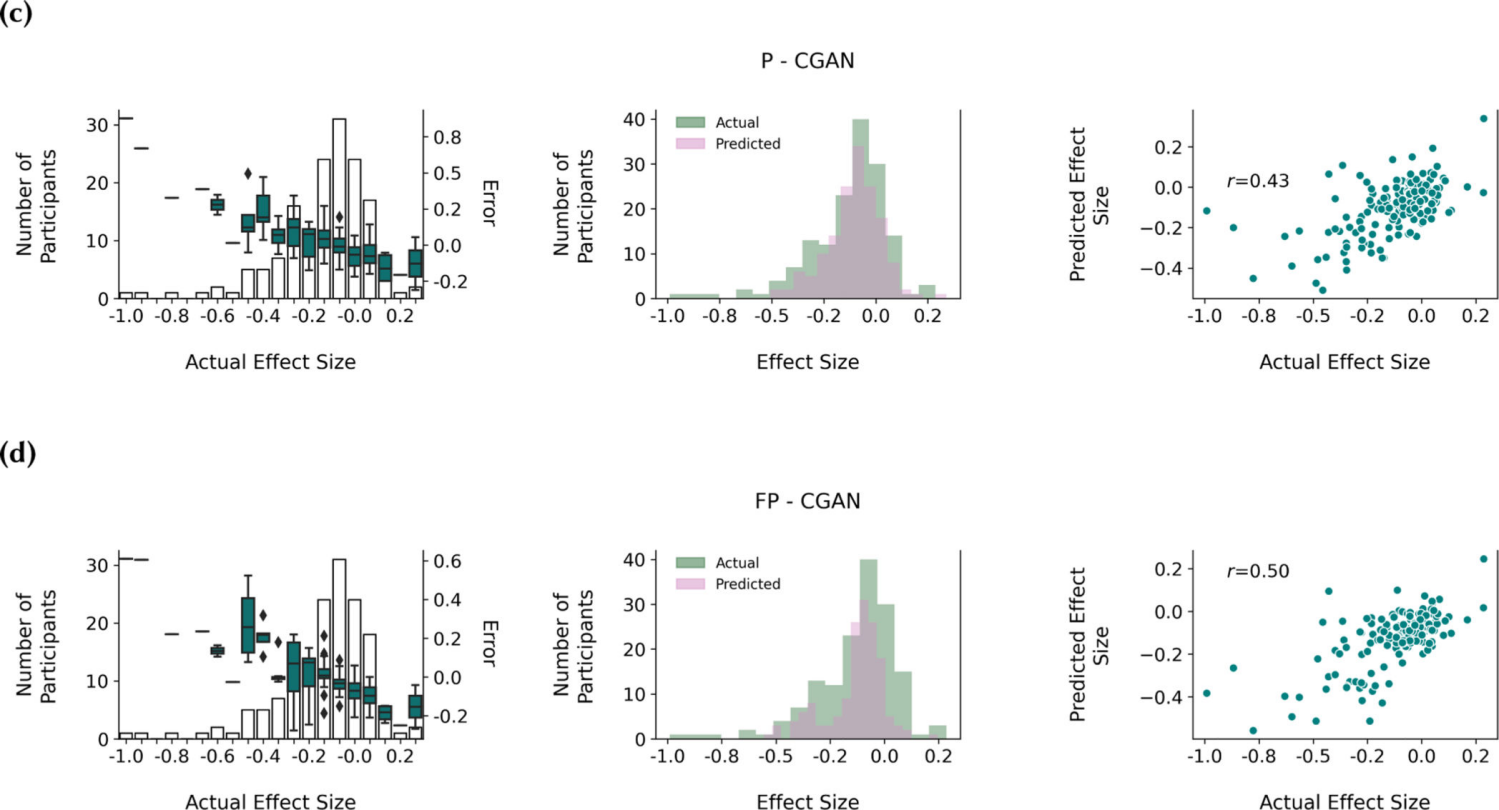
Comparing test set results across different models for the seconds of sleep per hour feature. **(a)** shows results for the participant multitasking multilayer perceptron (P - MLP) model, **(b)** the participant multitasking generator model (P GEN), **(c)** the participant multitasking conditional generative adversarial network (P - CGAN) model, and **(d)** the feature and participant multitasking conditional generative adversarial network (FP - CGAN) model. The left column plots show the error (predicted - actual) distributions between the individual-level actual and predicted Cohen’s *d_s_*. The boxplots overlay a histogram describing the number of participants whose actual Cohen’s *d_s_* fell into a designated range. Each box represents the error distribution for the participants within the underlying Cohen’s *d_s_* range. The middle column shows a histogram comparing the actual and predicted Cohen’s *d_s_*, and the right column shows this information in a scatterplot, where each point represents a test individual with the skipped correlation coefficient [103] values (*r*) labeled.

Figure 6 highlights differences in performance between the P - MLP, P - GEN, P - CGAN, and FP - CGAN models. The left column bar charts show that all models achieved better performance around the mode of the distribution because the boxplots, which represent the error between the predicted and actual Cohen’s *d_s_*, fall within higher magnitude error for individuals whose Cohen’s *d_s_* are on the tails of the distribution. The histograms in the middle column highlight that the CGAN models were able to predict a wider range of Cohen’s *d_s_* compared to both the P - MLP and P - GEN models, which only predicted values around the cluster modes. The right column scatterplots shows the improved prediction accuracy of the CGAN models at a participant-level.

### 6.4 Identifying Predicted Passive Sensing and EMA Indicators of Resilience

#### 6.4.1 GEE.

We performed the univariate GEE analysis described in section 4.3 with the predicted passive sensing and EMA indicators to explore if the predicted indicators also differentiated stress-resilient and stress-sensitive individuals. The indicators were calculated using internship data generated from the FP - CGAN model, and we used data from both the train and test sets for this analysis. Specifically, we focused on the indicators of resilience identified in section 4.3. The GEE results are found in Table 12. We did not include any indicators exclusive to the BL period, because they would be equivalent to what was shown in Table 9. Out of the 13 predicted indicators, 5 were significant. This included the seconds in bed skew during the internship, the mean heart rate during-internship, the mood EMA Cohen’s *d_s_*, mean, and standard deviation. The step count and seconds of sleep skew were marginally significant (*α* = 0.10). After conducting the univariate GEE, we conducted the same multivariate GEE described in section 4.3 using the predicted indicators. There were no significant indicators within the multivariate GEE.

#### 6.4.2 Comparing Actual and Predicted GEE Coefficients.

We then compared the coefficients of the significant predicted (Table 12) and actual (Table 9) passive sensing and EMA indicators associated with differentiating stress-resilient and stress-sensitive individuals. For this comparison, we concatenated the datasets containing the calculated actual and predicted features. We then created two variables: (1) a binary variable that dictated whether a given feature value was from the predicted or actual data, and (2) an interaction term between (1) and the feature values. We then used GEE with the same controls and specialty grouping to explore the associations between these two new features and the original feature for differentiating stress-resilient individuals. The interaction term coefficients modeled the change in the *β* coefficient when using the actual versus the predicted values for regression. If the coefficient was significant (*α* = 0.05), the difference between the actual and predicted coefficients were significantly different. We conducted this analysis for both the univariate GEE coefficients (*β*_*U*_) and multivariate (*β*_*M*_).

**Fig. 7.**
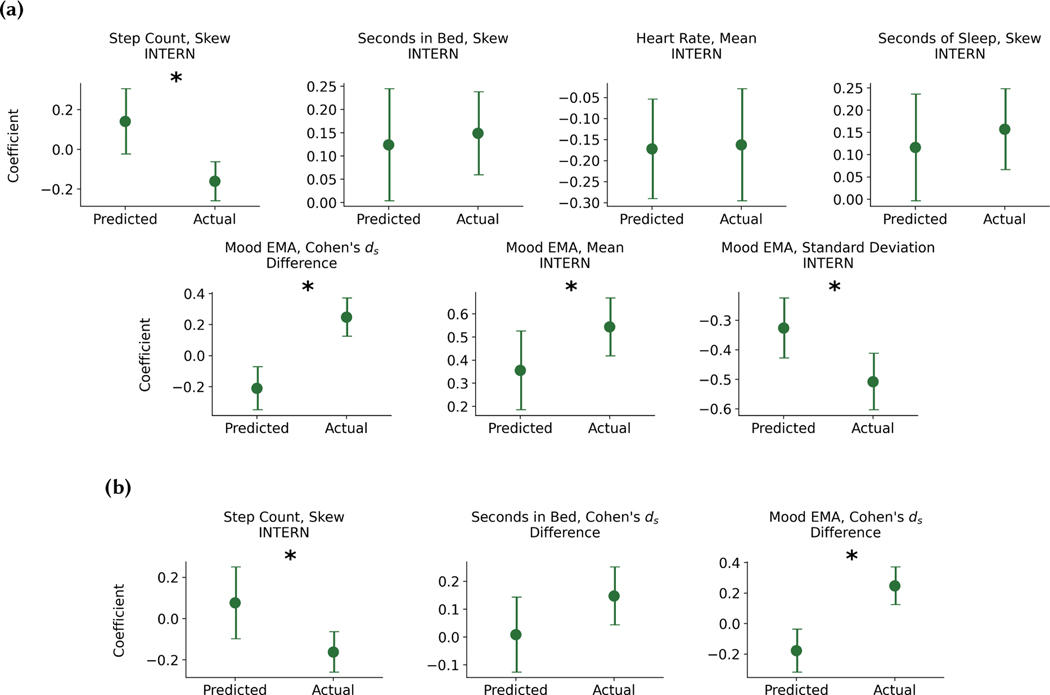
Plots of the shared significant coefficients from GEE using calculated features from the actual and predicted data. All features were either calculated using generated data from the internship (INTERN) or a difference between the internship and baseline periods (Difference). **(a)** shows the coefficient differences from the univariate and **(b)** from the multivariate GEE. There is a single plot per feature. The y-axis on each plot is the resulting *β* coefficient from conducting GEE to measure the effect of the feature from distinguishing stress-resilient versus sensitive individuals. The x-axis dictates whether the plotted values are from the GEE using the actual or predicted values. Points are the mean value of the coefficient, and error bars represent 95% confidence intervals. * indicates that the coefficients are significantly (*α* = 0.05) different.

Figure 7a shows the comparison between the actual and predicted coefficients for the univariate GEEs. We found 3 indicator coefficients were not significantly different. These included the seconds in bed skew, the mean heart rate, and the seconds of sleep skew during the internship. Figure 7b shows the comparison between the actual and predicted coefficients for the 3 multivariate GEE indicators. We found 1 feature coefficient that was not significantly different, specifically the seconds in bed Cohen’s *d_s_*.

## 7. DISCUSSION

In this work, we found indicators of resilience using passive sensing and EMA features, and then presented a novel method to predict these indicators. We then validated that the predicted and actual indicators showed the same associations with resilience. This discussion focuses on interpreting the research.

### 7.1 Implications

This research found specific passive and active sensing indicators of stress-resilience from mobile sensing data in a large (*n* = 775) population of medical interns. The associations between the indicators and stress-resilience are quantified in Table 9. These results show the potential for developing completely passive methods for measuring stress-resilience, and the mood EMA indicator shows that we may be able to develop less obtrusive active measures for monitoring resilience than repeated PHQ-9 measurements. These findings could help to identify both who may be most at risk for depression and, most importantly, provide an early signal of when they are most at risk. This knowledge can help inform more effective and targeted depression prevention and early detection strategies.

We also developed novel density estimation approaches that were able to approximate during-internship indicators. We then compared the associations between stress-resilience and the generated indicators to the original associations that existed between stress-resilience and the actual indicators. Figure 7 shows that we were able to generate data that held accurate associations between stress-resilience and the internship seconds in bed skew and Cohen’s *d_s_*, sleep duration skew, and average heart rate. The implication is that we can potentially anticipate sleep and heart rate changes that individuals may experience. We can then apply the found associations in Table 9 and translate these predicted changes into stress-related mental health symptoms early-on during the internship. Only three months of internship data are required to generate these accurate predictions, and the relationships to resilience they hold pertain to the entirety of the 12-month internship.

Based upon our findings, one might ask: if an individual were to change their sleeping patterns or partake in activities that lower average heart rate, would stress-resilience increase? This is a question that requires researchers to understand the causal associations between behavior and stress-resilience, either through conducting a randomized-control trial (RCT) with specific behavior change interventions, or applying methods such as propensity score matching [5] to observational data, which can estimate a treatment effect. Propensity score matching requires careful design of the study cohort such that all meaningful confounding variables are controlled for and the sample is representative of the true study population. This study, though large, was not representative (see Tables 6 and 7), and thus we are hesitant to conclude our associations are causal. Even with propensity score matching, an RCT is the gold-standard for measuring the treatment effect. The associations in Table 9 identify behavioral targets that can be incorporated into RCTs to measure how modulating these behavioral targets affect resilience. The findings in Figure 7 indicate the potential to design predictive interventions, which tested through RCTs, may anticipate behavioral changes and offset the future effects of prolonged stress.

### 7.2 Interpreting Mobile Sensing Indicators of Resilience

We found 17 mobile sensing features (Table 9) that were significantly associated with distinguishing stress-sresilient and sensitive individuals within the univariate GEE models. These indicators included information across all created hourly features. We now briefly discuss these indicators in more context, and point to literature to understand how they impact stress-resilience more broadly.

First, step count was intended as a proxy for physical activity. Physical activity mediates the effect of stress on impacting health [59, 77] and exercise can help individuals cope with stress [28]. We cannot state that the “step count” feature had a specific relationship to exercise, but we did find that individuals with less skew in their hourly step count distributions were more likely to be resilient. Since the step count distributions are right-skewed with a mode near 0, we can interpret this association to mean that individuals who spend more time walking tend to be resilient. On the other hand, increased heart rate can be associated with stress, and it was not surprising that a lower heart rate was associated with higher stress-resilience [67, 96].

Maintaining regular sleep habits has an effect on mediating depression symptoms [41, 46], and poor sleep quality is associated with increased stress [36] and stress-sensitivity [51]. Thus, it is not surprising that the 4 indicators we found significantly associated with sleep and resilience all showed that increased sleep increases the likelihood of resilience. One important note is that increased sleep duration does not necessarily translate to increased sleep quality, and quality is more associated with fatigue [72]. We had hypothesized that time in bed would have less of an effect on resilience than time spent sleeping, but the differences between these features within the GEE were minimal. Lastly, higher mood scores were associated with an increased likelihood of resilience, which is not surprising given many PHQ-9 questions are associated with mood [46]. In addition, lower mood variability was associated with an increased likelihood of resilience, and mood instability is associated with depression, anxiety, and post-traumatic stress disorder (PTSD) [10].

The multivariate GEE (see Table 9) revealed 3 indicators that were significantly associated with stress-resilience. These indicators had the same relationships with resilience as their equivalent univariate GEE associations, including a negative association with internship step count skew (*β*_*M*_ = −0.16), a positive association with increased time in bed (*β*_*M*_ = 0.11), and a positive association with increased mood (*β*_*M*_ = 0.26). These indicators all used data from the internship period, and thus showed that understanding the internship data and its relationship to resilience may be more important than using the baseline data alone.

### 7.3 Modeling Choices and Impact

#### 7.3.1 Identifying Mobile Sensing Indicators of Resilience.

We identified only 3 indicators of resilience in our multivariate GEE, compared to the 17 indicators of resilience identified within the univariate GEEs. The 3 indicators that remained in the multivariate GEE involved the internship step count skew, and the seconds in bed and mood EMA Cohen’s *d_s_*. The heart rate and seconds of sleep features were not included in the multivariate GEE, nor any indicators specific to the baseline period. This reduction could have been caused by our outlier filtering procedure, which may have homogenized our dataset and reduced differences between these indicators. In addition, we followed a strict method to reduce multicollinearity. The reduction of indicators showed that many indicators were correlated. This is expected for indicators such as seconds in sleep and in bed, where an increase in sleep increases time in bed. It also is reasonable when comparing indicators that are based upon a Cohen’s *d_s_* metric and the mean value, as modifying the mean value directly changes the Cohen’s *d_s_*. In the future, a richer multimodal dataset could be used to find more diverse associations to resilience without high multicollinearity.

#### 7.3.2 Predicting Mobile Sensing Indicators Using CGANs.

We developed novel CGAN models and applied these models to predict sensor and EMA data. Figure 6 highlights how using a conditional GAN (CGAN) and multitasking improved model performance for one example feature. Both the P - MLP and P - GEN models performed well around the modes of each participant multitasking cluster, but could not generate diverse Cohen’s *d_s_* even with participant multitasking, a problem that may be similar to mode collapse [85]. Using a CGAN improved the model’s ability to generate diverse samples, as the middle column in Figure 6 shows a greater match between the actual and predicted Cohen’s *d_s_* distributions in both the P - CGAN and FP - CGAN models. Feature multitasking without participant multitasking did not appear to create noticeable model improvements (Table 11). Future work should seek to understand why the CGAN architecture created more sample diversity compared to direct prediction and specific multitasking adjustments did or did not improve prediction performance.

That being said, all models still predicted a lower range of Cohen’s *d_s_* compared to the actual data (Figure 6). Underestimating the magnitude of the Cohen’s *d_s_* could have implications towards modeling stress-resilience if we expect individuals with larger mental health changes to also experience larger feature changes between the BL and INTERN periods. Future models can integrate re-sampling strategies to increase the prediction performance at the tails of the distribution.

We saw larger error variability across models for the seconds of sleep, in bed, and mean heart rate features (Table 11). Participant multitasking CGAN models appeared to improve the performance of predicting these features, and a qualitative error analysis reveals that the clusters (Figure 5) had more differentiated modes for these three features compared to the step count and mood EMA. The clusters for the participant multitasking models were created using the Cohen’s *d_s_*. We found 2 clusters, and the differences between these clusters can be found in Figure 5. We were surprised that there was not a greater amount of heterogeneity in our population. Our outlier filtering procedure, and the dropping of missing hours of data, may have reduced the population heterogeneity and contributed to the small number of clusters. In the future, more modeling work should be performed to analyze how outliers affect model performance.

#### 7.3.3 Analyzing the Relationships between Predicted and Actual Indicators of Resilience.

We found 5 significant and 2 marginally significant associations with resilience in the univariate GEEs using the predicted indicators of resilience, and analyzed their associations with coefficients from the same indicators using actual data (Figure 7a).predicted indicators produced coefficients that were not significantly different with the actual indicators. The predicted indicators that did produce significantly different coefficients included 3 indicators from the mood EMA and 1 indicator based upon the step count skew. The correlation between the actual and predicted mood EMA Cohen’s *d_s_* was smaller (*r* = 0.37) than other features, so it is understandable that the predicted mood had a different relationship with resilience than the actual mood data. That being said, for both the mood EMA mean and standard deviation, the predicted associations (*β*_*U*_ = 0.36 and *β*_*U*_ = −0.33) were in the same direction as the actual associations (*β*_*U*_ = 0.54 and *β*_*U*_ = −0.51) with stress-resilience, but the magnitude of the associations was less, potentially due to less variability within the GAN predictions.

The relationship between step count skew and resilience was positive in the predicted data, but negative in the actual data. There are a number of potential reasons why this association may be different. First, we did not directly optimize for the median value within the CGAN models, which is a component of the skew equation. Second, our models appeared to generate less diverse distributions than the actual data distributions (see Figure 6). Skew is a direct measure of extreme values within a dataset, and thus, if we were unable to generate extreme values, it is likely our generated distributions would be less skewed than the actual distributions.

The multivariate GEE using the predicted indicators showed 1 indicator whose coefficient was not significantly different than the multivariate GEE coefficients using the actual features and 2 that were (Figure 7b). The 2 coefficients that were significantly different were the step count count skew and mood EMA Cohen’s *d_s_*, and these differences are likely due to the same reasoning described above. The seconds in bed predicted and actual Cohen’s *d_s_* had the same association with resilience. It is important to note that the predicted indicator was not significant in the multivariate regression, so it is difficult to state whether the prediction follows the same association as the actual data, or the variation around the predicted coefficient happens to intersect with the actual coefficient value.

### 7.4 Ethics, Privacy, and Paths Forward

Passive sensing data can contain sensitive information, and individuals have expressed concerns with having this information collected for mental health monitoring [91]. This concern may be exacerbated among residents who already experience heightened perceived stigma for receiving mental health support [32]. Thus, we must be extremely careful when framing appropriate-use of technology to protect user-privacy and affirm the supportive role of passive data collection for improving resilience.

We propose that any intervention deploying our methods, or similar methods outlined in this paper should be codesigned by technologists and residents. Within this codesign process, ethical standards should be created by residents to articulate the capabilities and limitations of the technologies deployed, and these standards should become direct requirements of the intervention system. In addition, researchers should investigate integrating privacy-preserving machine learning methods into symptom monitoring models such that training and prediction can occur without individual-level data leaving a user’s device [1, 107]. The same approach could be used for collecting sensitive information from mobile devices to monitor mental health more broadly.

### 7.5 Limitations

The analyzed population in this work was not representative of medical school graduates by sex and race/ethnicity (see Table 6), and thus the results only apply to the analyzed cohort, and may not generalize beyond this cohort. Also, we used a specific measure of stress-resilience by analyzing PHQ-9 (depression symptom) changes over time, and labelled individuals who experienced minimal PHQ-9 changes as the “stress-resilient” population. Our results may differ if we chose to use trait resilience scales as a resilience measure, or a different mental health measure (eg, GAD-7). In addition, our held-out test dataset (*n* = 154) was large compared to other studies [97, 100] that have assessed feasibility for using mobile sensing data to model mental health, but a larger and more representative dataset would be needed to improve generalizability and approximate causal estimates.

The FP - CGAN model, which resulted in the best performance, required Q1 data for cluster matching. In addition, adherence to wearing the Fitbit and completing EMAs decreased across participants during the internship year, particularly between Q2 and Q3. Thus, the predicted passive sensing and EMA features were more specific to the first 6 months of the internship. We still believe this work is meaningful despite this limitation. Previous work [31] has found that by the end of Q2, mental health symptoms have peaked across a majority of interns. Most of our 775 participants (n=655, Table 3) had Q2 data, and we also found that the majority of participants’ (n=763, Figure 2) mental health symptoms peaked by the end of Q2.

In addition, we did not capture all features that could have been calculated from the Fitbit (eg, standard deviation of heart rate in an hour), and we did not collect EMAs that captured more information relevant to resilience other than mood. Future work should explore a richer feature space.

### 7.6 Future Work

Medical school graduates, who then begin their internship, are unequally represented across sex and race/ethnicity within the true population [65, 66]. Model performance may be inaccurate within specific demographic groups that are underrepresented even if the collected data is representative of medical school graduates. Future work should analyze whether the algorithms presented are biased in prediction due to underrepresentation and develop methods to reduce this bias.

GANs and stress-resilience models have not been rigorously studied in the context of survey and longitudinal behavioral and physiological data. Many of our design choices, including our interpolation of the mood EMA, adding noise to force mood EMA continuity, outlier filtering, and other data preprocessing procedures may have affected model performance. Follow-up work should be conducted to study the effects of these design choices on generative model performance using similar multivariate mobile sensing and survey data. We also wish to collect data that has better reliability, which would allow us to explore whether temporal features are associated with resilience. In addition, researchers could broaden the passive sensing feature set to capture other information about mental health. For example, prior studies have found that location-based [80, 81] and sleep regularity features [64] are associated with mental health symptoms.

Lastly, researchers should work with residents and other relevant program stakeholders to design interventions that use unobtrusive monitoring to improve mental health and well-being. While we have mentioned potential use cases that involve both the resident (eg, routine changes) and program administration (eg, increased schedule flexibility), it is difficult to design the appropriate effective intervention without engaging with residency programs.

### 7.7 Concluding Remarks

To the best of our knowledge, we created the first approach to identify and predict indicators of resilience using passive sensing data collected from Fitbit devices and EMA. We formulated novel generative adversarial networks (GANs) that applied multitask learning to predict behavioral, physiological, and well-being features associated with starting a medical internship, and validated if the predicted features could also be used as indicators of resilience. We hope this work adds a meaningful contribution to communities studying ubiquitous computing, generative modeling, and psychology, and paves the way forward for leveraging unobtrusive measurement for resilience-building interventions.

## Supplementary Material

Appendix

## Figures and Tables

**Table 1.** Passive sensing and EMA data collected during the Intern Health Study through the Fitbit and study application.

Data Type	Description
Heart rate	Heart rate each minute
Steps	Step count each minute
Sleep	The duration of sleep and short wake cycles, when the sleep event was recorded, as well as the category
Mood EMA	Question prompt: On a scale of 1 (low) to 10 (high), what was your average mood today?

**Table 2.** Passive sensing and EMA features used within this work.

Data type	Derived feature(s)
Heart rate	The hourly mean heart rate
Mood EMA	Interpolated daily self-reported EMA
Sleep	Time (in seconds) spent sleeping and in bed over an hour
Steps	Number of steps taken over an hour

**Table 3.** Summary of the hourly feature outlier filtering results. Outlier filtering was conducted using an Isolation Forest algorithm [52]. Results are listed in each cell using the following notation: (before filtering, after filtering). Some features, for example the seconds of sleep, have “0” values for the lower percentiles because individuals are not sleeping during most hours of the day. The mean heart unit is in beats per minute (BPM). Note that if sleep is continuous, the Fitbit may record one long multi-hour sleep cycle, but these cycles are usually broken-up by short wake cycles when someone moves while lying down or becomes restless.

Feature	Minimum	25th Percentile	Median	75th Percentile	Maximum
Step Count	(0, 0)	(0, 0)	(125, 135)	(495, 494)	(50017, 5496)
Seconds in Bed	(0, 0)	(0, 0)	(0, 0)	(600, 1320)	(89580, 8190)
Seconds of Sleep	(0, 0)	(0, 0)	(0, 0)	(120, 870)	(58920, 7740)
Mean Heart Rate (BPM)	(35, 35)	(63, 62)	(72, 71)	(82, 81)	(204, 130)
Mood EMA	(1, 1)	(7, 7)	(7, 7)	(8, 8)	(10, 10)

**Table 4.** Overview of the data filtering process, including the total number of participants, total hours of data, and the median (IQR) hours of data of across participants, split by baseline (BL) and each quarter (Q1–4). Each metric is listed for both the raw and cleaned data used for analysis. The median is the 50th percentile, and the IQR is a range representing the 25–75th percentiles of the data. Note that we enforced participants to have 100 hours of collected data in BL, and within the combined Q1–4, hence the median and 25th percentile increases in hours of data during certain periods after data cleaning. In addition, it is possible that participants may have dropped out of the study and returned, resulting in an increase in participants across specific periods of the study.

Period	Number of Participants		Total Hours of Data		Hours of Data Across Participants	
	Raw	Cleaned	Raw	Cleaned	Raw	Cleaned
BL	2,167	775	910,201	312,405	307 (28–634)	363 (237–532)
Q1	2,481	775	2,315,504	651,173	850 (20–1,850)	797 (408–1,220)
Q2	1,738	655	1,697,665	433,360	875 (60–1,867)	564 (205–1,060)
Q3	1,571	303	1,247,209	222,273	449 (18–1,667)	647 (275–1,130)
Q4	1,290	392	1,089,434	227,727	551 (20–1,719)	427 (155–939)
Total	2,668	775	7,260,013	1,846,938	495 (25–1,544)	508 (259–941)

**Table 5.** Overview of analyzed data.

	Analyzed Data
Total Participants, n	775
Total Hours of Data, n	1,846,938
Total Days of Data, n	116,536
Baseline Hours of Data per participant, median (IQR)	363 (237–532)
Internship Hours of Data per participant, median (IQR)	1,411 (554–2,974)

**Table 6.** Demographic information of the analyzed data compared to U.S. medical graduates from 2018–19 [65, 66]. The AAMC does not report the age of graduating medical students, and thus was excluded.

	Analyzed Data	U.S. Medical Graduates
Age, median (IQR)	27 (26–28)	Not reported
Female, n (%)	422 (55)	9,555 (48)
White, n (%)	505 (65)	10,879 (55)
Black, African American, n (%)	36 (5)	1,238 (6)
Hispanic, Latino, Spanish Origin, n (%)	25 (3)	1,063 (5)
Asian, n (%)	133 (17)	4,299 (22)
Native Hawaiian, Other Pacific Islander, n (%)	0 (0)	9 (<1)
American Indian, Alaskan Native, n (%)	0 (0)	38 (<1)
Mixed Race/Ethnicity, n (%)	63 (8)	1,598 (8)
Other Race/Ethnicity, n (%)	12 (1)	380 (2)
Race/Ethnicity Unlisted, n (%)	1 (<1)	124 (1)

**Table 7.** Intern medical specialties within the analyzed data.

Specialty	Analyzed Data, n (%)
Internal Medicine	173 (22)
Surgery	76 (10)
Ob/Gyn	50 (6)
Pediatrics	108 (14)
Psychiatry	35 (5)
Emergency Medicine	70 (9)
Med/Peds	25 (3)
Family Practice	70 (9)
Transitional	28 (4)
Anesthesiology (w/o transitional year)	36 (5)
Neurology (w/o transitional year)	13 (2)
Otolaryngology (w/o transitional year)	7 (1)
Other	84 (11)

**Table 8.** Results from using growth mixture models to identify different trajectories of depression symptom changes within our population. We used the Akaike Information Criterion (AIC) and the Bayesian Information Criterion (BIC) to assess model performance.

# of classes	AIC	BIC
2	17,267	17,318
3	17,184	17,254
4	17,127	17,215
5	17,135	17,242

**Table 9.** Results from conducting GEE to understand how each potential passive sensing and EMA indicator distinguishes stress-resilient and stress-sensitive individuals. Specialty was used as a grouping variable, and we controlled for sex and age in the model. We list only significant results (*α* = 0.05) from the univariate regressions. The *β*_*U*_ and *P*_*U*_ are the univariate significance and p-value respectively. Multivariate regression was performed after filtering out highly-correlated indicators. *β*_*U*_ and *P*_*M*_ are the coefficient and significance values for the 3 indicators included in the multivariate regression. Values were either specific to the period before (BL), during (INTERN) the internship, or captured a difference in a specific metric between the INTERN and BL periods (Difference).

Hourly Feature	Metric	Period	*β*_*U*_ (95% CI)	*P*_*U*_	*β*_*M*_ (95% CI)	*P*_*M*_
Step Count	Skew	BL	−0.19 (−0.37 to −0.01)	<.05		
Step Count	Skew	INTERN	−0.16 (−0.26 to −0.06)	<.01	−0.16 (−0.26 to −0.06)	<.01
Seconds in Bed	Cohen’s *d*_*s*_	Difference	0.15 (0.04 to 0.25)	<.01	0.11 (0.00 to 0.22)	<.05
Seconds in Bed	Mean	INTERN	0.17 (0.08 to 0.27)	<.001		
Seconds in Bed	Skew	INTERN	0.15 (0.06 to 0.24)	<.01		
Seconds in Bed	Standard Deviation	INTERN	0.13 (0.03 to 0.22)	<.05		
Heart Rate	Mean	BL	−0.13 (−0.25 to −0.01)	<.05		
Heart Rate	Mean	INTERN	−0.16 (−0.30 to −0.03)	<.05		
Seconds of Sleep	Cohen’s *d*_*s*_	Difference	0.15 (0.05 to 0.25)	<.01		
Seconds of Sleep	Mean	INTERN	0.18 (0.09 to 0.27)	<.001		
Seconds of Sleep	Skew	INTERN	0.16 (0.07 to 0.25)	<.001		
Seconds of Sleep	Standard Deviation	INTERN	0.13 (0.03 to 0.23)	<.01		
Mood EMA	Cohen’s *d*_*s*_	Difference	0.25 (0.12 to 0.37)	<.001	0.26 (0.13 to 0.39)	<.001
Mood EMA	Mean	BL	0.31 (0.14 to 0.48)	<.001		
Mood EMA	Mean	INTERN	0.54 (0.42 to 0.67)	<.001		
Mood EMA	Standard Deviation	BL	−0.23 (−0.33 to −0.12)	<.001		
Mood EMA	Standard Deviation	INTERN	−0.51 (−0.60 to −0.41)	<.001		

**Table 10.** Overview of the data used for model training and validation (80% of the total data), as well as the held-out data (20%) used to report model results.

	Training (80%)	Testing (20%)
Participants, n	611	154
Hours of data, n	1,452,667	394,271
Days of data, n	91,804	24,732

**Table 11.** Skipped correlation coefficient [103] (*r* ∈ [−1, 1]) values and significance between the predicted and actual individual-level Cohen’s *d_s_* for each model and feature.

Model	Step Count	Seconds of Sleep	Seconds in Bed	Mean Heart Rate	Mood EMA
MLP	0.27 **	0.23 **	−0.05	0.13	0.33 ***†
P - MLP	0.22 **	−0.18 *†	0.11 †	−0.05	0.34 ***
GEN	0.32 ***†	0.14	0.08	0.07	0.37 ***
P - GEN	0.46 ***	0.15 †	0.08 †	0.20 *	0.35 ***
CGAN	0.12	0.02	0.00	0.15	0.41 ***
F - CGAN	0.38 ***	0.12	0.09	0.15	0.35 ***
P - CGAN	0.19 *	0.43 ***†	0.42 ***†	0.24 **	0.36 ***
FP - CGAN	0.31 ***	0.50 ***†	0.49 ***†	0.21 *	0.37 ***

* *P* < .05,

** *P* < .01,

*** *P* < .001.

† indicates significant (*α* = 0.05) values within a Wilcoxon signed-rank test, testing the hypothesis that the squared error between the actual and predicted Cohen’s *d_s_* is less than the squared error achieved from assigning the mean Cohen’s *d_s_* from the training distribution to each individual. MLP = baseline multilayer perceptron regression model. GEN = baseline density estimation models. CGAN = conditional generative adversarial network. P = participant multitasking, F = feature multitasking.

**Table 12.** Results from conducting a univariate GEE using each indicator of resilience identified in Table 9 calculated from the predicted distributions. The GEE modeled the relationship between predicted passive sensing and EMA indicators and stress-resilience, with intern specialty as a grouping variable, and controlling for age and sex. *β*_*U*_ is the coefficient value, and *P*_*U*_ is the significance level. Indicators exclusive to the baseline (BL) period are not shown because they would have the equivalent *β*_*U*_ coefficient and significance level from Table 9. Predicted values were either specific to during the internship (INTERN), or captured a difference in a specific metric between the INTERN and BL periods (Difference).

Hourly Feature	Metric	Period	*β*_*U*_ (95% CI)	*P_U_*
Step Count	Skew	INTERN	0.14 (−0.02 to 0.30)	<.1
Seconds in Bed	Cohen’s *d_s_*	Difference	0.07 (−0.05 to 0.19)	
Seconds in Bed	Mean	INTERN	0.10 (−0.03 to 0.22)	
Seconds in Bed	Skew	INTERN	0.12 (0.00 to 0.24)	<.05
Seconds in Bed	Standard Deviation	INTERN	0.04 (−0.08 to 0.15)	
Heart Rate	Mean	INTERN	−0.17 (−0.29 to −0.05)	<.01
Seconds of Sleep	Cohen’s *d_s_*	Difference	0.07 (−0.06 to 0.19)	
Seconds of Sleep	Mean	INTERN	0.09 (−0.03 to 0.21)	
Seconds of Sleep	Skew	INTERN	0.12 (−0.00 to 0.24)	<.1
Seconds of Sleep	Standard Deviation	INTERN	0.04 (−0.07 to 0.15)	
Mood EMA	Cohen’s *d_s_*	Difference	−0.21 (−0.35 to −0.07)	<.01
Mood EMA	Mean	INTERN	0.36 (0.18 to 0.53)	<.001
Mood EMA	Standard Deviation	INTERN	−0.33 (−0.43 to −0.22)	<.001
