# Supplementary material for: Identifying Mobile Sensing Indicators of Stress-Resilience
Source: Proc ACM Interact Mob Wearable Ubiquitous Technol. Author manuscript; Available in PMC 2022 Apr 19. (PMC9017954; doi:10.1145/3463528)
Supplement: Appendix [file NIHMS1738299-supplement-Appendix.pdf]

APPENDIX TO: IDENTIFYING MOBILE SENSING INDICATORS OF STRESS-RESILIENCE

Permission to make digital or hard copies of part or all of this work for personal or classroom use is granted without fee provided that copies are not made or distributed for profit or commercial advantage and that copies bear this notice and the full citation on the first page of the published paper. Copyrights for third-party components of this work must be honored. For all other uses, contact the Owner/Author (dadler@infosci.cornell.edu).
©2021 Copyright held by the owner/author(s).

I ADDITIONAL DETAILS ON IDENTIFYING INDICATORS OF RESILIENCE

GEE parameters, like many linear models, cannot be reliably estimated as a multivariate regression if independent variables are highly correlated [1]. We suspected high correlations within the created 37 potential indicators of resilience, and thus we conducted our analysis in steps to reduce multicollineariaty. We first created separate GEE models for each potential indicator ("univariate GEE"), with controls and specialty groupings. We then filtered to significant ($\alpha = 0.05$) indicators from the univariate GEEs, and began adding each of these indicators to a combined "multivariate GEE" model. Indicators were added by iterating through each hourly feature, and significance. For example, we first would attempt to add the mood EMA most significant indicator, then the seconds in bed most significant indicator, etc. We used this ordering to create more hourly feature diversity within the multivariate GEE model. To reduce multicollinearity, indicators were only added to the multivariate GEE if their variance inflation factor (VIF) between the current indicators within the multivariate GEE was <5. VIF estimates the explained variance for an independent variable from all other independent variables within a model [3]. A VIF>5 means that more than 50% of a variable's variance is explained by all other independent variables currently within a model.

II DENSITY ESTIMATION MODELS

Figure II.1a gives an overview of the density estimation procedure. We now describe this procedure in more detail.

II.1 Generative Adversarial Networks (GAN)

II.1.1 Conceptual. GANs take an adversarial approach to density estimation. An adversarial approach is a type of modeling approach where two models compete against each other [4]. In a GAN, the two models are called a generator, G, which is a neural network, and a discriminator, D, which is a second neural network. The generator's role is to input random noise, $z \in \mathcal{R}$, and generate a data point from this random noise that comes from a target distribution B. In other words, $G(z)$ outputs a $b' \in \mathcal{R}$, and b' comes from the generated distribution B'. As the generator is trained, ideally, $b' \longrightarrow b$, where $b \in B, b \in \mathcal{R}$ which in turn pushes $B' \longrightarrow B$.

The discriminator's (D) role is to take a generated (b') or real (b) data point, and discriminate between whether the data point comes from the real (B) or generated (B') distribution. In other words, the discriminator is a classifier that outputs a probability that an input comes from the real probability distribution. Thus, a perfect discriminator would output that $D(b) = 1$ and $D(b') = 0$. A GAN is called a generative "adversarial" network, because it is the generator's job to "fool" the discriminator into thinking that a generated data point is a true data point, which amounts to the discriminator outputting that a generated data point (b') is highly likely to come from the "real" distribution, i.e. $D(b') \longrightarrow 1$.

The generator, G, and discriminator, D, are jointly trained, which means they compete during model training. As the generator outputs higher quality data points, the discriminator learns to discriminate these higher quality data points, and thus the generator must re-learn to fool the discriminator. This "competition" continues during model training, and the generator learns to create data points that look more realistic.

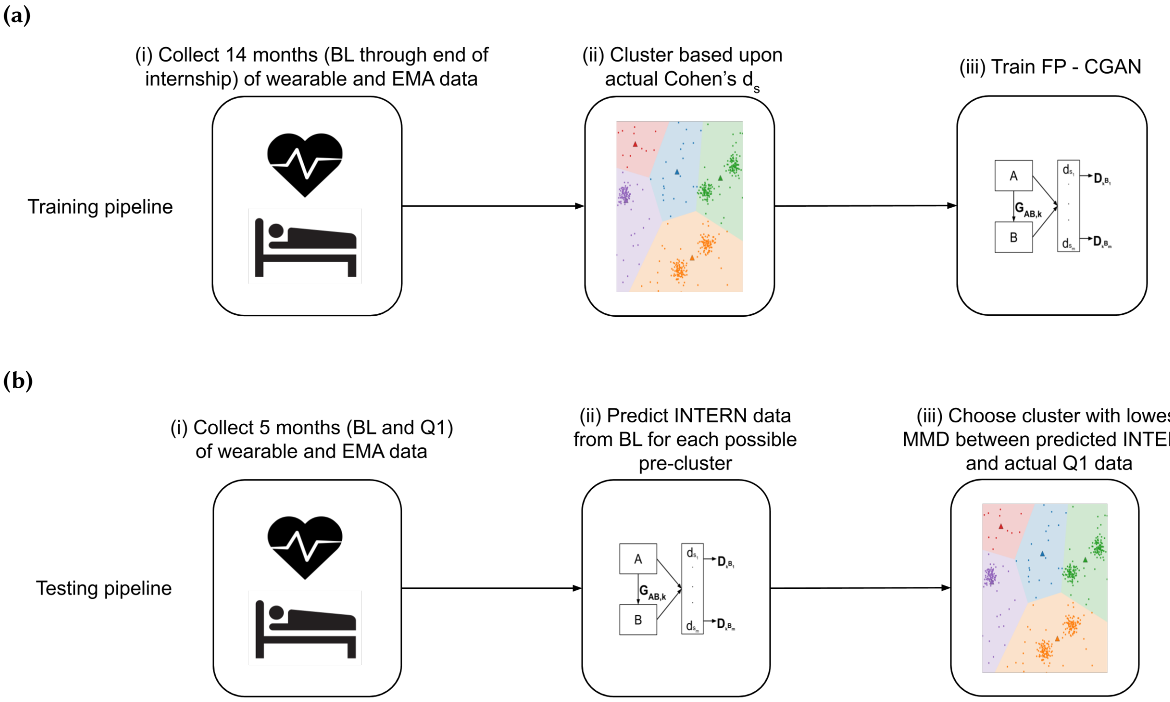

Fig. II.1. The **(a)** training and **(b)** testing pipeline. In the training pipeline **(a)** (from left to right), we collect 14 months of data from medical interns, use this data to create clusters (tasks) for participant multitasking models, and then train models that generate during-internship multivariate passive sensing and EMA densities per participant. In the testing pipeline **(b)** (from left to right), we collect data through the first quarter of the internship, and use this data to generate a multivariate density of passive sensing and EMA data per participant for the entirety of the internship. If the trained model uses participant multitasking, we generate data for each cluster (task), and choose the cluster whose generated data most closely matches the actual Q1 data of the participant.

II.1.2 Formalization. We can formalize this training process into equations. Let $z \in Z, z \in \mathcal{R}$ be a random noise scalar sampled from a standard normal distribution. Let $G : \mathcal{R} \longrightarrow \mathcal{R}$ be a generator, which creates a generated $b' \in B', b' \in \mathcal{R}$ from a random z, i.e. $b' = G(z)$. Let $D : \mathcal{R} \longrightarrow \mathcal{R}$ be a discriminator, or a classifier, that outputs a probability that an input data point comes from the true data distribution B.

We can thus define an objective function, similar to the original GAN formulation in [4]. Since the discriminator wants to distinguish real data points with high probability, it wants to maximize $D(b)$ and minimize $D(b') = D(G(z))$. This is equivalent to the discriminator maximizing both $D(b)$ and $1-D(b') = 1-D(G(z))$. The generator wants to maximize $D(G(z))$, or minimize $1 - D(G(z))$ which is equivalent to "fooling" the discriminator. The optimization occurs by training the following equation:

$$min_G max_D \mathcal{L}_{GAN}(G, D, B, Z)$$

$$\mathcal{L}_{GAN}(G, D, B, Z) = \mathbb{E}_{b \in B}[D(b)^2] + \mathbb{E}_{z \in Z}[(1 - D(G(z)))^2] \quad (1)$$

$$b \in B, z \in Z, b \in \mathcal{R}, z \in \mathcal{R}$$

The objective includes both $\mathbb{E}_{b \in B}[D(b)^2]$ and $\mathbb{E}_{z \in Z}[(1 - D(G(z)))^2]$, where $\mathbb{E}[\cdot]$ is the expected value, or mean of a distribution. Qualitatively, this translates to optimizing the mean squared error of discriminator output using both actual data points within B and generated $G(Z) = B'$.

II.1.3 Conditional GAN. In this work, we wanted to generate a specific multivariate hourly feature internship distribution B from a multivariate hourly feature baseline distribution A per participant. There is a family of GANs, called conditional GANs (CGAN), that add an additional loss to the GAN objective described in equation 1. The objective of this conditional loss is to push the generated distribution for a participant, B', to the actual target internship distribution B, per participant.

We followed a similar CGAN framework as [6]. Let us define multivariate data points $a \in A, b \in B, a \in \mathcal{R}^m, b \in \mathcal{R}^m$, where A is the multivariate baseline distribution and B is the multivariate intern distribution for a participant. m is the number of hourly features. We now define a multivariate generator, $G_{AB} : \mathcal{R}^m \longrightarrow \mathcal{R}^m$, such that $b' = G_{AB}(a)$. Thus, the generator inputs one multivariate hourly baseline data point, and outputs a generated internship multivariate hourly data point. We also define a multivariate discriminator $D_B : \mathcal{R}^m \longrightarrow \mathcal{R}$. The discriminator inputs an internship multivariate real or generated hourly data point, b or b', and outputs the likelihood the data point comes from an actual or generated distribution. The GAN objective used in this work is the following two-player game.

$$min_{G_{AB}} max_{D_B} \mathcal{L}_{GAN}(G_{AB}, D_B, A, B)$$

$$\mathcal{L}_{GAN}(G_{AB}, D_B, A, B) = \mathbb{E}_{b \in B}[D_B(b)^2] + \mathbb{E}_{a \in A}[(1 - D_B(G_{AB}(a)))^2] \quad (2)$$

$$a \in A, b \in B, a \in \mathcal{R}^m, b \in \mathcal{R}^m$$

We also introduced a conditional loss, or $\mathcal{L}_{CON}$, similar to [6], which attempts to minimize the differences between the actual $b \in B$ and generated $b' \in B'$ for each participant. Within this work, specific data points $a \in A$ and $b \in B$ are unpaired in the sense that there is no specific a that should directly map to a b. That being said, a well-generated distribution for an individual, B', should have the same characteristics (eg, mean, variance, skewness) as the actual distribution B. We thus wanted to choose a conditional loss function focused on high-level distribution characteristics instead of trying to minimize the error between individual generated and actual data points.

The maximum mean discrepancy (MMD) is a two sample test, testing the hypothesis that two samples are drawn from the same distribution [5]. The MMD has been used to measure the differences between a true and generated data distribution from a GAN [13]. The MMD compares the differences between a kernel estimated over the individual actual and generated distributions, and the mixed distribution of actual and generated data. The MMD approaches 0 as the sum of the individual kernels approach the mix (i.e. the distributions become equivalent). Again, let A be a set of multivariate hourly feature data points used to generate B' for a specific participant. Let $k(x, y)$ be a kernel function. We can define the MMD conditional loss as follows:

$$\mathcal{L}_{CON}(G_{AB}, A, B) = \mathbb{E}_{\substack{a,a^* \in A \\ b,b^* \in B}} \left[k\big(G_{AB}(a), G_{AB}(a^*)\big) + k(b, b^*) - 2k\big(G_{AB}(a), b\big) \right]$$

$$a \in A, a^* \in A, b \in B, b^* \in B, a \in \mathcal{R}^m, a^* \in \mathcal{R}^m, b \in \mathcal{R}^m, b^* \in \mathcal{R}^m \tag{3}$$

Similar to previous work [9], we used the kernel function $k(x, y) = \sum_{q=1}^{K} k'_{\sigma_q}(x, y)$ where $k'_{\sigma_q}(x, y)$ is a radial basis function (RBF) and σ_q is an adjustable bandwidth parameter. We let σ_q equal $\{1, 2, 4, 8, 16\}$. The full objective and loss can be thus described as

$$min_{G_{AB}} max_{D_B} \mathcal{L}(G_{AB}, D_B, A, B)$$

$$\mathcal{L}(G_{AB}, D_B, A, B) = \mathcal{L}_{GAN}(G_{AB}, D_B, A, B) + \mathcal{L}_{CON}(G_{AB}, A, B) \tag{4}$$

$$a \in A, b \in B, a \in \mathcal{R}^m, b \in \mathcal{R}^m$$

II.1.4 Cohen's d_s Discriminator. We created a novel CGAN framework specifically for modeling changes in the passive sensing and EMA data that can be used to predict indicators of resilience. To do this, we had the discriminator operate on a calculated feature space, namely the hourly feature Cohen's d_s. We expected that the raw A and B distributions would overlap, and thus having the GAN loss operate on the raw feature space would confuse the optimization algorithm. By having the discriminator operate on the Cohen's d_s, we would give better feedback to the GAN loss.

The GAN loss can be re-written as the following, where $d_s \in \mathcal{R}^m$ is a vector of Cohen's d_s across m features. We will define $d_s^{A,B}$ as the Cohen's d_s that uses the multivariate actual internship data (B), and $d_s^{A,G_{AB}}$ as the Cohen's d_s that uses the multivariate generated internship data (G_{AB}). Note that we are no longer taking the expectation over the data, since the Cohen's d_s summarizes the baseline and generated distribution changes:

$$\mathcal{L}_{GAN}(G_{AB}, D_B, A, B) = \left[D_B\big(d_s^{A,B}\big)\right]^2 + \left[1 - D_B\big(d_s^{A,G_{AB}}\big)\right]^2$$

$$a \in A, b \in B, a \in \mathcal{R}^m, b \in \mathcal{R}^m, d_s^{A,B} \in \mathcal{R}^m, d_s^{A,G_{AB}} \in \mathcal{R}^m \tag{5}$$

The model with the Cohen's d_s discriminator will be referred to as the "CGAN" for the rest of this work, and the process to input and output a multivariate hourly data point into the CGAN can be found in Figure II.2. A higher level view of the CGAN can be found in Figure II.3a.

II.1.5 Multitask CGAN. Multitask learning (MTL) is a machine learning technique used to train separate, but related prediction tasks together [2]. Previous work has used MTL to train separate but related facial detection tasks, such as face pose estimation and facial localization [14], and has also improved neural network model performance within tasks that individually have scarce data [7]. We experimented with two novel applications of MTL within a CGAN. We did not have enough data to train a task for each individual. Similar to [12], we clustered individuals together that experienced similar feature changes once the internship began, and treated training a model for each cluster as a separate task. We will describe the clustering procedure in future sections.

We consider a multivariate hourly data point from the internship distribution composed of m features $b = (b_1, ..., b_m), b \in \mathcal{R}^m$. We will treat the process to train a network to accurately generate a distribution of each internship hourly feature $b_j \in B_j, b_j \in \mathcal{R}$ as a single task. We first created a *Feature Multitask CGAN* (F - CGAN), described in Figure II.3b, where we utilized the CGAN as a base model, but then replaced the single multivariate discriminator for all features D_B with a separate discriminator, $D_{B_j} : \mathcal{R} \rightarrow \mathcal{R}$ for each feature that is trained to

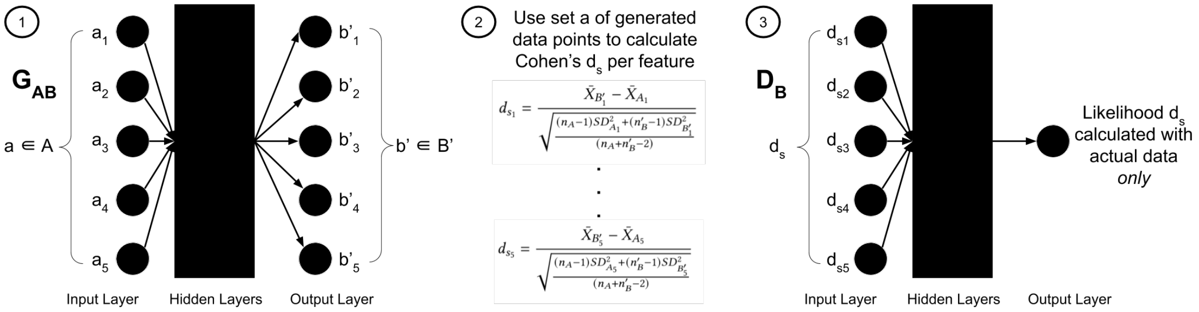

Fig. II.2. The conditional generative adversarial network (CGAN) architecture. Hidden layers are shown as black boxes for simplicity, but they are composed of multiple fully connected neural network layers. As an example, we describe how a multivariate data point can be generated and passed into the discriminator. (1) A single multivariate baseline hourly data point $a \in A$ is input into the generator, G_{AB}, which outputs a single generated multivariate hourly internship data point, $b' \in B'$. (2) After inputting a set of multivariate baseline data points into the generator and outputting a set of generated internship data points for an individual, a generated internship multivariate mean $\bar{X}_{B'_j}$ and sample standard deviation $SD_{B'_j}$ can be calculated for each feature, $j, j \in \{1, ..., m\}$, where there exist m total features. This can be used to then calculate a predicted Cohen's d_s for each feature, $d_{s_1}, ..., d_{s_m}$. (3) The Cohen's d_s for each feature can be input into the multivariate input layer of the discriminator D_B, which outputs the probability the multivariate Cohen's d_s was calculated using actual or generated data. For the feature multitasking networks, the discriminators for each feature's Cohen's d_{s_j} do not share any layers. For the participant multitasking networks, there are additional output layers specific to each cluster on the generator, and each cluster has a unique discriminator which does not share any layers with other cluster discriminators.

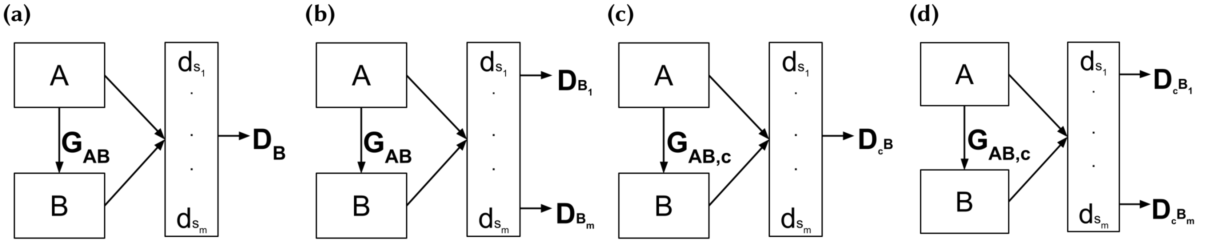

Fig. II.3. The **(a)** conditional generative adversarial network (CGAN) which uses a discriminator with Cohen's d_s inputs, **(b)** feature multitask learning CGAN (F - CGAN) with separate discriminators (D_{B_j}) for each feature's Cohen's d_{s_j} **(c)** participant multitask learning CGAN (P - CGAN) with separate output layers for each cluster in the generator ($G_{AB,c}$) and separate discriminators per clusters (D_{cB}) **(d)** and feature participant multitask learning CGAN (FP - CGAN), which combines the separate feature and cluster discriminators (D_{cB_j}), as well as cluster-specific hidden layers in the generator ($G_{AB,c}$). A is a multivariate hourly feature baseline distribution for an individual, and B is a multivariate hourly feature internship distribution for an individual. G_{AB} is a generator, and D_B is a discriminator. $j \in \{1, ..., m\}$ identifies a specific feature, out of m total features, and $c \in \{1, ..., C\}$ is a participant cluster out of C possible clusters. d_{s_j} is the Cohen's d_s for a specific feature, calculated from baseline and internship data.

predict whether a feature's Cohen's d_{s_j}, is an actual or predicted Cohen's d_{s_j}. Let $(G_{AB})_j$ be a specific feature output from a multidimensional generator G_{AB}. The new GAN loss then averages together the individual GAN losses from the separate discriminators:

$$\mathcal{L}_{GAN} = \frac{1}{m}\sum_{j=1}^{m} \mathcal{L}_{jGAN}((G_{AB})_j, D_{B_j}, A_j, B_j)$$

$$\mathcal{L}_{jGAN}((G_{AB})_j, D_{B_j}, A_j, B_j) = \left[D_{B_j}\left(d_{s_j}^{A_j,B_j}\right)\right]^2 + \left[1 - D_{B_j}\left(d_{s_j}^{A_j,(G_{AB})_j}\right)\right]^2 \tag{6}$$

$$b_j \in B_j, a_j \in A_j, b_j \in \mathcal{R}, a_j \in \mathcal{R}, d_{s_j} \in \mathcal{R}, (G_{AB})_j \in \mathcal{R}, j \in \{1, ..., m\}$$

We also created a *Participant Multitask CGAN* (P - CGAN), described in Figure II.3c, where we first clustered participants based upon their actual Cohen's d_s, and treated each cluster as a single task. Specifically, we added an extra linear layer to the generator, G_{AB}, and trained a separate discriminator for each cluster. If we have C clusters, we would thus train C separate D_B. During training, we can pick a specific participant, and only back-propagate through the shared layers and cluster-specific layers for that participant. In this case, let c be the cluster for a participant, and let $G_{AB,c}$ be a generator that will propagate an input through an extra linear layer specifically for cluster c. Let D_{cB} be the discriminator for cluster c, and d_s the multidimensional Cohen's d_s. The GAN loss becomes:

$$\mathcal{L}_{GAN}(G_{AB,c}, D_{cB}, A, B) = \left[D_{cB}\left(d_s^{A,B}\right)\right]^2 + \left[1 - D_{cB}\left(d_s^{A,G_{AB,c}}\right)\right]^2$$

$$b \in B, a \in A, b \in \mathcal{R}^m, a \in \mathcal{R}^m, d_s \in \mathcal{R}^m, c \in \{1, ..., C\} \tag{7}$$

Finally, we combined both the F - CGAN and P - CGAN to create a *Feature and Participant - Multitask CGAN* (FP - CGAN), described in Figure II.3d, which first propagated a cluster-specific output for a participant, and then had separate discriminators for each cluster and feature, D_{cB_j}. The FP - CGAN $\mathcal{L}_{GAN}$ loss is:

$$\mathcal{L}_{GAN} = \frac{1}{m}\sum_{j=1}^{m} \mathcal{L}_{jGAN}((G_{AB,c})_j, D_{cB_j}, A_j, B_j)$$

$$\mathcal{L}_{jGAN}((G_{AB,c})_j, D_{cB_j}, A_j, B_j) = \left[D_{cB_j}\left(d_{s_j}^{A_j,B_j}\right)\right]^2 + \left[1 - D_{cB_j}\left(d_{s_j}^{A_j,(G_{AB,c})_j}\right)\right]^2 \tag{8}$$

$$b_j \in B_j, a_j \in A_j, b_j \in \mathcal{R}, a_j \in \mathcal{R}, d_{s_j} \in \mathcal{R}, (G_{AB,c})_j \in \mathcal{R}, c \in \{1, ..., C\}, j \in \{1, ..., m\}$$

II.2 Clustering for Participant MTL Models

We clustered the training data using K-Means and Ward Hierarchical Agglomerative Clustering [8] to find initial participant clusters (individual MTL tasks) within the participant MTL models. The Cohen's d_s for each training participant and feature were calculated, and we used principle components analysis to reduce noise within the feature space. The silhouette score, which quantifies both the tightness of within-cluster data and the distance between adjacent clusters, was used to choose the number of components, clusters, and clustering algorithm. We varied the number of clusters from 2-10, and we added component dimensions until 99% of the variance between features was explained. After model training, we generated data from all clusters for each test participant. We

then computed the MMD between the generated data, and the actual first quarter internship data for each test participant. The generated data from the cluster that achieved the lowest MMD per participant compared to the first quarter data (Q1) were included within our results, and all other data were removed for that participant.

II.3 Model Hyperparameters

Models were built using Pytorch [10], and trained for 1,000 epochs. During each epoch, we trained models using a modified batch gradient descent procedure to force model updates to account for individual participant differences, and act as a regularizer. We iterated through every training participant during each epoch, and randomly sampled n_{batch} data points per participant where $n_{batch} = min(n_A, n_B)$, defining n_A, n_B as the number of hourly data points within individual-level distributions A, B respectively. Gradient updates were performed during every participant by epoch iteration. Models were trained using the Adam optimizer with different initial learning rates (0.001, 0.0001).

All generators and discriminators used fully connected linear layers within an encoder-decoder based architecture similar to [6], but with fully connected layers only. Given m input features to a network, the generator architecture had three hidden layers of size $(2m, 4m, 2m)$, and the discriminator architecture had two hidden layers of size $(2m, 4m)$. In addition, we trained a neural network model (GEN) that optimized only the generator G_{AB}, to study if using the CGAN framework improved the prediction performance over a simpler model. We also applied participant multitasking to the baseline generator model (P - GEN) to see if it improved baseline model performance. We experimented with adding more hidden layers across models, but deeper networks increased training time with minimal improvements to model performance.

Dropout (rate = 0.2) [11] was used for additional regularization between linear layers of the discriminator. All features were scaled between $[-1, 1]$. ReLU activation was used for the generator hidden layers and Tanh activation was used for the output activation. Leaky ReLU layers were used for the discriminator hidden activation (negative slope = 0.2), and a sigmoid layer was used for the output activation.

The model architecture and training procedure were chosen based upon experimentation within the training dataset, by examining the effects of changes in these parameters on convergence and runtime. After solidifying the model architecture and training procedure, all models were trained using the 80% training data, and then predictions were calculated for held-out test participants, for each model and initial learning rate.

II.4 Initial Learning Rates

We trained models with three initial learning rates (0.01, 0.001, 0.0001), and for space, reported the results for each model with the initial learning rate that minimized the median MMD across participants on the held-out test dataset. The initial learning rates for these final models are reported in Table II.1.

REFERENCES

[1] Aylin Alin. 2010. Multicollinearity. *WIREs Computational Statistics* 2, 3 (2010), 370–374. https://doi.org/10.1002/wics.84 _eprint: https://onlinelibrary.wiley.com/doi/pdf/10.1002/wics.84.

[2] Rich Caruana. 1997. Multitask Learning. *Machine Learning* 28, 1 (July 1997), 41–75. https://doi.org/10.1023/A:1007379606734

[3] Trevor A. Craney and James G. Surles. 2002. Model-Dependent Variance Inflation Factor Cutoff Values. *Quality Engineering* 14, 3 (March 2002), 391–403. https://doi.org/10.1081/QEN-120001878 Publisher: Taylor & Francis Ltd.

[4] Ian J. Goodfellow, Jean Pouget-Abadie, Mehdi Mirza, Bing Xu, David Warde-Farley, Sherjil Ozair, Aaron Courville, and Yoshua Bengio. 2014. Generative Adversarial Networks. (June 2014). https://arxiv.org/abs/1406.2661v1

[5] Arthur Gretton, Karsten M. Borgwardt, Malte J. Rasch, Bernhard Schölkopf, and Alexander Smola. 2012. A Kernel Two-Sample Test. *Journal of Machine Learning Research* 13, 25 (2012), 723–773. http://jmlr.org/papers/v13/gretton12a.html

[6] Phillip Isola, Jun-Yan Zhu, Tinghui Zhou, and Alexei A. Efros. 2018. Image-to-Image Translation with Conditional Adversarial Networks. *arXiv:1611.07004 [cs]* (Nov. 2018). http://arxiv.org/abs/1611.07004 arXiv: 1611.07004.

Table II.1. Initial learning rates used for each model within the main manuscript.

Model	Learning Rate
P - MLP	0.01
MLP	0.001
GEN	0.0001
P - GEN	0.0001
CGAN	0.0001
F - CGAN	0.001
P - CGAN	0.001
FP - CGAN	0.001

[7] Feng Jin and Shiliang Sun. 2017. Neural Network Multitask Learning for Traffic Flow Forecasting. *arXiv:1712.08862 [cs]* (Dec. 2017). http://arxiv.org/abs/1712.08862 arXiv: 1712.08862.

[8] Joe H. Ward Jr. 1963. Hierarchical Grouping to Optimize an Objective Function. *J. Amer. Statist. Assoc.* 58, 301 (March 1963), 236–244. https://doi.org/10.1080/01621459.1963.10500845 Publisher: Taylor & Francis _eprint: https://www.tandfonline.com/doi/pdf/10.1080/01621459.1963.10500845.

[9] Chun-Liang Li, Wei-Cheng Chang, Yu Cheng, Yiming Yang, and Barnabás Póczos. 2017. MMD GAN: Towards Deeper Understanding of Moment Matching Network. *arXiv:1705.08584 [cs, stat]* (Nov. 2017). http://arxiv.org/abs/1705.08584 arXiv: 1705.08584.

[10] Adam Paszke, Sam Gross, Francisco Massa, Adam Lerer, James Bradbury, Gregory Chanan, Trevor Killeen, Zeming Lin, Natalia Gimelshein, Luca Antiga, Alban Desmaison, Andreas Köpf, Edward Yang, Zach DeVito, Martin Raison, Alykhan Tejani, Sasank Chilamkurthy, Benoit Steiner, Lu Fang, Junjie Bai, and Soumith Chintala. 2019. PyTorch: An Imperative Style, High-Performance Deep Learning Library. *arXiv:1912.01703 [cs, stat]* (Dec. 2019). http://arxiv.org/abs/1912.01703 arXiv: 1912.01703.

[11] Nitish Srivastava, Geoffrey Hinton, Alex Krizhevsky, Ilya Sutskever, and Ruslan Salakhutdinov. 2014. Dropout: A Simple Way to Prevent Neural Networks from Overfitting. (2014), 30.

[12] Sara Ann Taylor, Natasha Jaques, Ehimwenma Nosakhare, Akane Sano, and Rosalind Picard. 2017. Personalized Multitask Learning for Predicting Tomorrow's Mood, Stress, and Health. *IEEE Transactions on Affective Computing* (2017), 1–1. https://doi.org/10.1109/TAFFC.2017.2784832

[13] Qiantong Xu, Gao Huang, Yang Yuan, Chuan Guo, Yu Sun, Felix Wu, and Kilian Weinberger. 2018. An empirical study on evaluation metrics of generative adversarial networks. *arXiv:1806.07755 [cs, stat]* (Aug. 2018). http://arxiv.org/abs/1806.07755 arXiv: 1806.07755.

[14] Cha Zhang and Zhengyou Zhang. 2014. Improving multiview face detection with multi-task deep convolutional neural networks. In *IEEE Winter Conference on Applications of Computer Vision*. 1036–1041. https://doi.org/10.1109/WACV.2014.6835990 ISSN: 1550-5790.
